# Mitochondrial inorganic polyphosphate is required to maintain proteostasis within the organelle

**DOI:** 10.3389/fcell.2024.1423208

**Published:** 2024-07-10

**Authors:** Renata T. Da Costa, Pedro Urquiza, Matheus M. Perez, YunGuang Du, Mei Li Khong, Haiyan Zheng, Mariona Guitart-Mampel, Pia A. Elustondo, Ernest R. Scoma, Vedangi Hambardikar, Beatrix Ueberheide, Julian A. Tanner, Alejandro Cohen, Evgeny V. Pavlov, Cole M. Haynes, Maria E. Solesio

**Affiliations:** ^1^ Department of Biology, College of Arts and Sciences, Rutgers University, Camden, NJ, United States; ^2^ Department of Molecular, Cell, and Cancer Biology, University of Massachusetts Chan Medical School, Amherst, MA, United States; ^3^ School of Clinical Medicine, LKS Faculty of Medicine, The University of Hong Kong, Pokfulam, Hong Kong SAR, China; ^4^ School of Biomedical Sciences, LKS Faculty of Medicine, The University of Hong Kong, Pokfulam, Hong Kong SAR, China; ^5^ Center for Advanced Biotechnology and Medicine, Rutgers University, New Brunswick, NJ, United States; ^6^ Biological Mass Spectrometry Core Facility, Faculty of Medicine, Dalhousie University, Halifax, NS, Canada; ^7^ Proteomics Laboratory, Division of Advanced Research Technologies, New York University-Grossman School of Medicine, New York City, NY, United States; ^8^ Materials Innovation Institute for Life Sciences and Energy (MILES), HKU-SIRI, Shenzhen, China; ^9^ Advanced Biomedical Instrumentation Centre, Hong Kong Science Park, Hong Kong SAR, China; ^10^ Department of Molecular Pathobiology, College of Dentistry, New York University, New York City, NY, United States

**Keywords:** mitochondria, mitochondrial inorganic polyphosphate, polyP, protein homeostasis, proteostasis

## Abstract

The existing literature points towards the presence of robust mitochondrial mechanisms aimed at mitigating protein dyshomeostasis within the organelle. However, the precise molecular composition of these mechanisms remains unclear. Our data show that inorganic polyphosphate (polyP), a polymer well-conserved throughout evolution, is a component of these mechanisms. In mammals, mitochondria exhibit a significant abundance of polyP, and both our research and that of others have already highlighted its potent regulatory effect on bioenergetics. Given the intimate connection between energy metabolism and protein homeostasis, the involvement of polyP in proteostasis has also been demonstrated in several organisms. For example, polyP is a bacterial primordial chaperone, and its role in amyloidogenesis has already been established. Here, using mammalian models, our study reveals that the depletion of mitochondrial polyP leads to increased protein aggregation within the organelle, following stress exposure. Furthermore, mitochondrial polyP is able to bind to proteins, and these proteins differ under control and stress conditions. The depletion of mitochondrial polyP significantly affects the proteome under both control and stress conditions, while also exerting regulatory control over gene expression. Our findings suggest that mitochondrial polyP is a previously unrecognized, and potent component of mitochondrial proteostasis.

## 1 Introduction

Increased mitochondrial protein misfolding and aggregation is a well-known trigger of mitochondrial dysfunction, which ultimately leads to increased apoptotic cell death in many human pathologies (Nakamura et al., 2012). Despite the situation in other organelles, such as the endoplasmic reticulum (ER) (Araki and Nagata, 2012), the mechanism that governs mitochondrial mammalian proteostasis still remains largely unknown (Haynes and Ron, 2010; Pellegrino et al., 2013; Rainbolt et al., 2013; Schulz and Haynes, 2015). One of the main intracellular contributors to protein dyshomeostasis is the increased generation of reactive oxygen species (ROS). ROS are mostly generated in mitochondria during oxidative phosphorylation (OXPHOS); hence mitochondrial proteins are especially prone to be deleteriously affected by ROS. Moreover, it is known that misfolded proteins are imported from the cytosol into mitochondria, probably for degradation (Ruan et al., 2017). All this evidence suggests the presence of a potent mechanism that regulates protein homeostasis within the organelle. However, this mechanism is poorly understood.

Inorganic polyphosphate (polyP) is a well-conserved throughout evolution and ubiquitous polymer (Morrissey et al., 2012; Osorio et al., 2022). It is composed of multiple subunits of orthophosphates linked together by phosphoanhydride bonds, similar to those present in ATP (Kornberg et al., 1999; Muller et al., 2017; Desfougeres et al., 2020). While polyP shows a ubiquitous distribution among cells and organisms, a high presence of this polymer in mammalian mitochondria has already been demonstrated (Abramov et al., 2007; Solesio et al., 2016a). In fact, the regulatory effects of polyP on the maintenance of cellular bioenergetics are known in mammals and other organisms (Solesio et al., 2016a; Solesio et al., 2020; Borden et al., 2021; McIntyre and Solesio, 2021; Solesio et al., 2021; Scoma et al., 2023). Maintaining proper protein homeostasis is a highly energy-dependent process. Therefore, the close relationship between the status of OXPHOS and that of mitochondrial proteostasis has already been demonstrated (Nargund et al., 2015). Accordingly, the regulatory role of polyP on protein homeostasis has been proposed in different organisms. For example, polyP is a primordial chaperone in bacteria, and this effect is independent of any transcriptional factors (Gray et al., 2014). Furthermore, the important role played by polyP in the regulation of amyloidogenesis is also known (Cremers et al., 2016; Yoo et al., 2018; Lempart and Jakob, 2019; Xie and Jakob, 2019). This effect is induced by the ability of the polymer to accelerate fibril formation of amyloidogenic proteins, and it protects against amyloid-induced cytotoxicity (Lempart et al., 2019). Aging is one of the main trigger of protein dyshomeostasis, including amyloidogenesis, in mammals (Squier, 2001). Interestingly, polyP levels decrease with aging in these organisms (Lorenz et al., 1997).

The main goal of our study was to address the regulatory role of mitochondrial polyP in proteostasis within the organelle. To conduct our experiments, we used Wild-type (Wt) and MitoPPX HEK293 cells. MitoPPX cells have been enzymatically depleted of mitochondrial polyP. We previously thoroughly characterized them (Guitart-Mampel et al., 2022; Hambardikar et al., 2022), demonstrating that MitoPPX HEK293 cells have decreased endogenous levels of mitochondrial polyP. Using this model, our data show the protective role of mitochondrial polyP against heat shock (HS)-induced protein aggregation within the organelle. Moreover, ourpull-down data supports previous findings regarding the ability of polyP to bind to proteins in mammals (Labberton et al., 2018), and it show that the specific proteins bound to polyP are different under control and stress conditions. Lastly, our data demonstrate the strong regulatory effects exerted by mitochondrial polyP on the expression levels of some genes, and the levels of some proteins, involved in the maintenance of protein homeostasis within the organelle, in response to ROS-induced stress.

Our data indicate a potent regulatory role for mitochondrial polyP in the maintenance of proper protein homeostasis within the organelle, especially under stress conditions, and independently of the nature of the stress stimuli. While mitochondrial protein dyshomeostasis has been broadly described in human pathology, the molecular mechanisms involved in this process remain to be fully elucidated. Therefore, our findings could contribute to the understanding of the etiopathology of many of these diseases. These conditions, for example, include all the main neurodegenerative disorders (Irvine et al., 2008; Patro et al., 2021).

## 2 Materials and methods

### 2.1 Reagents

Dulbecco’s Modified Eagle medium (DMEM), penicillin/streptomycin, geneticin (G418), trypsin, heat-inactivated fetal bovine serum (FBS), and MitoTracker Green were purchased from Gibco-Invitrogen (Carlsbad, CA, United States). Dimethyl Sulfoxide (DMSO), toluidine blue (TBO), rotenone, Triton X-100, sodium chloride (NaCl), methanol, phosphate-buffered saline (PBS), β-mercaptoethanol, tris(hydroxymethyl)-1,3-propanediol hydrochloride (TRIS-HCl), glycerol, phenylmethylsulfonyl fluoride (PMSF), Tween-20, potassium chloride (KCl), sucrose, mannitol, ethylenediaminetetraacetic acid (EDTA), ethylene glycol-bis(2-aminoethylether)-N,N,N′,N′-tetraacetic acid (EGTA), acetic acid, lodoacetamide, 1,4-dithiothreitol (DTT), formaldehyde, deuterated formaldehyde, triethylammonium bicarbonate buffer (TEAB), sodium cyanoborohydride, urea, calcium chloride, trifluoroacetic acid, acetonitrile, formic acid, ammonium formate, ammonium hydroxide, ammonium bicarbonate, LiCl, Ziptips, and acetone were purchased from Sigma-Aldrich (St. Louis, MO, United States). 1-ethyl-3-(3-dimethylaminopropyl) carbodiimide (EDAC), Pierce BCA protein assay kit, Pierce ECL Western blotting substrate, NuPAGE 4%–12% Bis-Tris Gel, P700-immobilised beads, Poros beads, GelCode Blue Strain, and Pierce halt protease and phosphatase inhibitor cocktails were purchased from Thermo Fisher Scientific (Waltham, MA, United States). Alexa Fluor 647 cadaverine was purchased from Life Technologies (Carlsbad, CA, United States).

### 2.2 Cell culture

Human Embryonic Kidney (HEK293) cells were acquired from the American Type Culture Collection (ATCC, Manassas, VA, United States). MitoPPX HEK293 cells were created as previously described (Solesio et al., 2016b; Solesio et al., 2021). Briefly, MitoPPX cells were generated by the stable transfection of HEK293 cells with a DNA construct containing the sequence for mammalian expression of the *Saccharomyces cerevisiae* exopolyphosphatase (PPX) enzyme, a mitochondrial-targeted sequence, and the sequence for expression GFP. Cells were grown in DMEM supplemented with 10% (v/v) FBS, 0.1 mg/mL streptomycin and 100 units/mL penicillin at 37°C and 5% CO_2_ in a humidified cell culture incubator. In the case of the MitoPPX cells, the medium was also supplemented with 40 μg/mL G418. The cells were maintained in a medium without G418 for at least 7 days prior to conducting the experiments. We have already broadly characterized MitoPPX HEK293 cells. For example, we have previously demonstrated that the mitochondrial expression of PPX is a valid method to decrease polyP levels in the organelle (Hambardikar et al., 2023). Moreover, our previous studies show that 48 h post seeding, cell viability of MitoPPX cells is not significantly different from that of Wt cells, under control conditions (Hambardikar et al., 2022). However, we have reported significant differences in the mitochondrial ultrastructure of MitoPPX cells; as well as in their ability to maintain proper bioenergetics, including the regulation of calcium homeostasis (Solesio et al., 2016a; Solesio et al., 2020; Solesio et al., 2021; Guitart-Mampel et al., 2022; Hambardikar et al., 2022; Hambardikar et al., 2023).

### 2.3 Rotenone treatment

Cells were plated on Petri dishes until they were at a 70%–80% confluence and treated with 4 or 6 µM rotenone for 3 h in fresh medium. After that, cells were harvested and the specific experiments were conducted.

### 2.4 LDH cell viability assay

Cells were seeded in 96-wells plates. After 48 h, they were treated with 4 or 6 µM rotenone. Triplicate wells were prepared for each experimental condition. LDH assay was then conducted following the instructions provided by the manufacturer (Roche, Indianapolis, IN, United States). Absorbance was measured at 492 and 620 nm using a microplate reader (CLARIOstart spectrophotometer, BMG Labtech, Ortenberg, Germany). Data was standardized vs. Wt control (0% cytotoxicity).

### 2.5 Immunoblotting assays

Immunoblotting assays were conducted as previously reported (Solesio et al., 2013a; Baltanas et al., 2013; Solesio et al., 2013b; Liu et al., 2019; Castro et al., 2020). Briefly, cell pellets were lysed for 15 min at 4°C with rotation in the presence of 50–100 μL of 1x RIPA buffer supplemented with protease inhibitor cocktail (Sigma-Aldrich, St. Louis, MO, United States). After centrifugate the samples at 14,000 xg for 5 min, the supernatant was transferred to a fresh 1.5 mL tube. Protein concentration of the cell lysates was determined by a BCA assay. 1× Laemmli Sample Buffer (BioRad, Hercules, CA, United States), which was completed with 2-Mercaptoetanol was added, and samples were incubated for 5 min at 95°C. Proteins were separated on 12% pre-cast acrylamide gels (BioRad, Hercules, CA, United States). Gels were then transferred into Polyvinylidene difluoride (PVDF) membranes (BioRad, Hercules, CA, United States) and blocked for 1 h in a solution containing 5% fat free milk and 0.05% Tween 20 in 1× PBS. Membranes were then incubated with the primary antibodies overnight at 4°C, followed by PBS washes and incubation with horseradish-peroxidase (HRP)-conjugated secondary antibodies for 1 h at room temperature. Chemiluminescent detection of bands was performed using ECL reagent (ThermoFisher Scientific, Waltham, MA, United States). Quantification of the bands was performed using ImageJ (NIH, Bethesda, MD, United States).

Primary antibodies were purchased from the following vendors: anti-βactin (Abcam, cat. ab8226); anti-CHOP (Cell Signaling, cat. 2895); anti-Sirt3 (Cell Signaling, cat. 2627); anti-SOD2 (Millipore, cat. 06-984); anti-Hsp60 (Santa Cruz, cat. sc-13115); anti-Hsp10 (Sigma-Aldrich, cat SAB4501465); anti-FoxO3a (Cell Signaling, cat. 2497); anti-Acetyl-FoxO3a (Affinity, cat. AF3771); anti-TOM20 (Cell Signaling, cat. 42406); anti-Parkin (Santa Cruz Technology, cat. sc-32282); and anti-LC3B (Abcam, cat. Ab192890); All the antibodies were used at a 1:1,000 dilution. The HRP-conjugated secondary antibodies used were anti-mouse; DαMo-HRP (Bio-Rad, cat. 170616), anti-Rabbit; DαRb-HRP (Bio-Rad, cat. 170615). In this case, both antibodies were used at a 1:5,000 dilution.

### 2.6 Mitochondrial isolation

Mitochondrial isolation was conducted as previously described (Fossati et al., 2016; Solesio et al., 2018; Solesio et al., 2021). Freshly isolated mitochondria were used for all the experiments.

### 2.7 HS experiments

HS was performed on isolated mitochondria. Specifically, mitochondria used for these experiments were placed for 30 min at 45°C. These conditions have been broadly used to induce HS in mammalian cells and to conduct protein biology studies (Duncan and Hershey, 1984; Mizzen and Welch, 1988; Abe et al., 1999; Kim et al., 2011). The control samples were maintained at 25°C for the same length of time. To maintain the desired temperatures, the tubes containing mitochondria were placed on a Mixer HC thermoblock (USA Scientific, Ocala, FL, United States).

### 2.8 Soluble and insoluble protein fractions preparation

After mitochondrial isolation and the corresponding treatments, protein content was quantified using the BSA assay. All the samples were then diluted to the lowest concentration found in each set of experiments, to ensure that equal amounts of protein were used to conduct the separation of the soluble and insoluble protein fractions. This separation procedure was carried out according to the previously described method (Wilkening et al., 2018). After the isolation of soluble and insoluble protein fraction, these fractions were loaded in gels and samples were analyzed via immunoblotting assay. Please, note that no loading controls were included since different amounts of proteins were loaded in each of the fractions (soluble and insoluble), due to the nature of the experiment. As mentioned above, the amount of protein used before starting the cellular fractioning was exactly the same. The obtained results are therefore qualitative, rather than quantitative.

### 2.9 Transcript expression analysis (RT-qPCR assay)

RNA was isolated from Wt and MitoPPX cells using the RNeasy Mini Kit (Qiagen, Hilden, Germany), following the protocol provided by the manufacturer. RNA concentrations were determined by spectrophotometry (NanoDrop One, GE Health Care, Buckinghamshire, United Kingdom). cDNA was synthetized using the QuantiTect Reverse Transcription Kit (Qiagen, Hilden, Germany), with an input of 0.5 µg of total RNA. The RT-qPCR amplifications were conducted using a QuantStudio™ 6 Flex Real-Time PCR System (Applied Biosystems, Foster City, CA, United States), and Power SYBR™ Green PCR Master Mix (Qiagen, Hilden, Germany). Specifically, we used the following thermal conditions: 10 min at 95°C (hot start); 45 cycles of 15 s at 95°C, and 25 s at 60°C. The primers were designed with Primer3web 4.1.0 (available at https://primer3.ut.ee/), and their sequences are described below. GAPDH (glyceraldehyde 3-phosphate dehydrogenase) was used as the housekeeping gene. The fold change values for the differential gene expression were evaluated using the 2^−ΔΔCt^ formula (Livak and Schmittgen, 2001).

**Table:** Primers used for the transcript expression analysis by RT-qPCR.

Gene	Forward and reverse sequences	Amplicon (bp)
GAPDH	TTG​GCT​ACA​GCA​ACA​GGG​TG	161
	GGG​GAG​ATT​CAG​TGT​GGT​GG	
SOD2	TGG​GGT​TGG​CTT​GGT​TTC​AA	95
	GGA​ATA​AGG​CCT​GTT​GTT​CCT​TG	
SIRT3	CGG​CTC​TAC​ACG​CAG​AAC​ATC	225
	CAG​CGG​CTC​CCC​AAA​GAA​CAC	
PRKN	GTG​CCG​TAT​TTG​AAG​CCT​CA	123
	GAC​AGG​GCT​TGG​TGG​TTT​TC	
TOMM20	CCC​CAA​CTT​CAA​GAA​CAG​GC	185
	GAT​GGT​CTA​CGC​CCT​TCT​CA	
DNM1L	AGA​ATA​TTC​AAG​ACA​GTG​TGC​CA	145
	TGT​GCC​ATG​TCC​TCA​GAT​TCT	
ATF5	CTG​GGA​TGG​CTC​GTA​GAC​TA	258
	CTT​GAG​GAG​GGA​GGC​CAT​AG	
HSPD1	GGG​TAC​TGG​CTC​CTC​ATC​TC	140
	CAC​TGT​TCT​TCC​CTT​TGG​CC	
HSPE1	CTT​TGC​GGC​GCT​ACA​CTA​G	118
	GCA​GCA​CTC​CTT​TCA​ACC​AA	
DDIT3	TTC​CTT​TTG​TCT​ACT​CCA​AGC​CTT​C	274
	ATG​AAA​GGA​AAG​TGG​CAC​AGC​TA	

### 2.10 Liquid chromatography-tandem mass spectrometry (LC-MS/MS)

We conducted LC-MS/MS assays in two distinct sets of samples: 1) Soluble and insoluble mitochondrial fractions from Wt and MitoPPX cells, under control conditions and after HS, and 2) gel pull-down of proteins bound to polyP. In each case, the sample preparation and the LC-MS/MS assay were conducted using different methods. In the case of 1), after the statistical “analysis” predictions of the affected pathways and diseases were created using Ingenuity Pathway Analysis (IPA, Qiagen, Hilden, Germany).

#### 2.10.1 Mitochondrial soluble and insoluble fractions

Cells were grown, scraped, and pelleted following the protocols described above. Mitochondria were isolated and HS was conducted. The protein content was quantified using the BCA assay, following the protocol described above. Subsequently, the soluble and insoluble fractions were separated as previously described, with equal amounts of protein used. The samples were then loaded onto SDS-PAGE gels and bands were excised as a single gel slice, following the previously published protocol (Sleat et al., 2008; Sleat et al., 2009). Subsequently, samples were treated as it follows:

##### 2.10.1.1 In-gel digestion

Each gel band was subjected to reduction with 10 mM DTT for 30 min at 60°C, followed by alkylation with 20 mM iodoacetamide for 45 min at room temperature in the dark. The bands were then digestion with trypsin and incubated overnight at 37°C.

##### 2.10.1.2 Peptide extraction

The peptides were extracted twice with a 5% formic acid and 60% acetonitrile. The resulting extract was then dried under vacuum.

##### 2.10.1.3 LC-MS/MS

Samples were analyzed by nano LC-MS/MS using an Eclipse tribid mass spectrometer interfaced with an Ultimate 3000 RSLCnano chromatography system (Thermo Fisher Scientific, Waltham, MA, United States). The samples were loaded onto a fused silica trap column (Acclaim PepMap 100, 75 μm × 2 cm, Thermo Fisher Scientific, Waltham, MA, United States). After washing for 5 min at a flow rate of 5 μL/min with 0.1% trifluoroacetic acid, the trap column was brought in-line with an analytical column (Nanoease MZ peptide BEH C18, 130 A, 1.7 μm, 75 μm × 250 mm, Waters, Milford, MA, United States) for LC-MS/MS analysis. Peptides were chromatographed at 300 nL/min using a segmented linear gradient 4%–15% B in 30 min (A: 0.2% formic acid; B: 0.16% formic acid, 80% acetonitrile), 15%–25% B in 40 min, 25%–50% B in 44 min, and 50%–90% B in 11 min.

The scan sequence began with an MS1 spectrum (Orbitrap analysis, resolution 120,000, scan range from M/Z 375–1,500, automatic gain control (AGC) target 1E6, maximum injection time 100 m). The top S (3 s) duty cycle scheme was used for determining the number of MSMS performed for each cycle. Parent ions of charge 2–7 were selected for MSMS and dynamic exclusion of 60 s was used to avoid repeat sampling. Parent masses were isolated in the quadrupole with an isolation window of 1.2 m/z, automatic gain control (AGC) target 1E5, and fragmented with higher-energy collisional dissociation with a normalized collision energy of 30%. The fragments were scanned in Orbitrap with resolution of 15,000. The MSMS scan ranges were determined by the charge state of the parent ion but lower limit was set at 110 atomic mass units.

##### 2.10.1.4 Database search

Peak lists (mgf files) were generated using Thermo Proteome Discoverer v. 2.1 (Thermo Fisher Scientific, Waltham, MA, United States) and searched against the Uniprot human database and a database composed of common contaminants (cRAP), using a local implementation of TANDEM (Craig and Beavis, 2004). The search parameters were as it follows: protein and peptide log10 expectation scores < −4 and −2, respectively; fragment mass error: 20 ppm, parent mass error: ± 7 ppm; static modification: Cys carboxymethylation; dynamic or variable modifications during the initial search: Gly-Gly tag on internal lysine for ubiquitination and monoxidation on methionine; dynamic or variable modifications during refinement: round 1, Met and Trp mono oxidation, Asn and Gln deamidation; round 2, Met and Trp dioxidation. The peptide false positive rates were 0.4%.

#### 2.10.2 Pull-down of proteins bound to polyP

Mitochondria were isolated from mice liver following the protocol published in (PMID: 22509855). Mice liver were obtained from animals used for other experiments in the department. Subsequently, functional mitochondria were heat shocked also as stated above, or treated with 200 μM H_2_O_2_ for 30 min. Following this, incubation with P700-immobilized beads, pull-down, elution using LiCl, and SDS-PAGE were conducted according to the previously published protocol (Khong et al., 2020). P-700 beads were used for the pull-down procedure, as preliminary studies using P-100 beads resulted in a minimal number of proteins being pulled-down.

##### 2.10.2.1 Gel digestion

Samples were reduced with 2 μL of 0.2 M DTT at 57°C for 1 h; alkylated with 2 μL of 0.5 M iodoacetic acid at room temperature in the dark for 45 min; and loaded onto a NuPAGE 4%–12% Bis-Tris Gel 1.0 mM. The gels were run for approximately 12 min at 200 V. Subsequently, the gel was then stained using GelCode Blue Stain Reagent and Coomassie-stained bands were excised from the gel for analysis.

The excised bands were first distained in a 1:1 v/v solution of methanol and 100 mM ammonium bicarbonate, and then partially dehydrated with acetonitrile and further dried using a SpeedVac for 20 min. Next, 200 ng of trypsin was added to each sample. After the enzyme was absorbed, 70–150 μL of 10 mM ammonium bicarbonate was added to cover the gel fragments. Finally, the gel fragments were digested overnight with agitation at room temperature.

##### 2.10.2.2 Protein extraction

The following day, R2 20 μM Poros beads in a solution of 5% formic acid and 0.2% trifluoroacetic acid were added to each sample in a 1:1 v/v proportion to the amount of ammonium bicarbonate added the previous night. The samples were soaked for 3 h at 4°C. Afterwards, the beads were loaded onto equilibrated C18 Ziptips and a microcentrifuge was used to spin them for 30 s at 6,000 xg. Gel fragments were rinsed three times with 0.1% trifluoracetic acid, and each rinse was added to its corresponding Ziptip, followed by microcentrifugation. The extracted Poros beads were then washed with 0.5% acetic acid. To elute the peptides, 40% acetonitrile in 0.5% acetic acid was used, followed by 80% acetonitrile in 0.5% acetic acid. The organic solvents were subsequently removed using a Speed Vac. Finally, the samples were then reconstituted in 0.5% acetic acid.

##### 2.10.2.3 Mass spectrometry

The samples were individually analyzed as previously described (Drummond et al., 2020). The spectra were searched against UniProt database using Byonic Search Engine within Proteome Discoverer.

### 2.11 Statistical analysis

All the experiments, including the samples for the proteomics assays, were performed at least in biological triplicates. The only exception to this is the experiment for the pull-down for proteins bound to polyP, which were conducted once, following the standard procedures in the field (Khong et al., 2020; Okpara et al., 2022). In the case of the IPA analysis, only pathways with −log (*p*-value) higher than 1.3 (that is, *p-*value ≤0.05) were included. Data is expressed as the mean ± SEM, unless otherwise stated. The relative units are expressed with respect to the control samples. Statistical analyses were carried out with GraphPad Prism (GraphPad, La Jolla, CA, United States). The comparison of the data was carried out using unpaired Student’s t-tests, one-way ANOVAs with Tukey’s *post hoc* analyses; and Brown-Forsythe and Welch ANOVA tests for data sets that the assumption of equal variances across groups was violated (stated in the figure legend). Values of *p* ≤ 0.05 were considered significant (*p ≤ 0.05, **p ≤ 0.01, ***p ≤ 0.001, ****p ≤ 0.0001). Please, note that in the parts of the graphs where nothing is indicated, *p* is higher than 0.05. Images of the original uncropped gels are included at the end of the Supplementary Material.

## 3 Results

### 3.1 Mitochondrial polyP prevents aggregation of TUFM after HS

**FIGURE 1:**
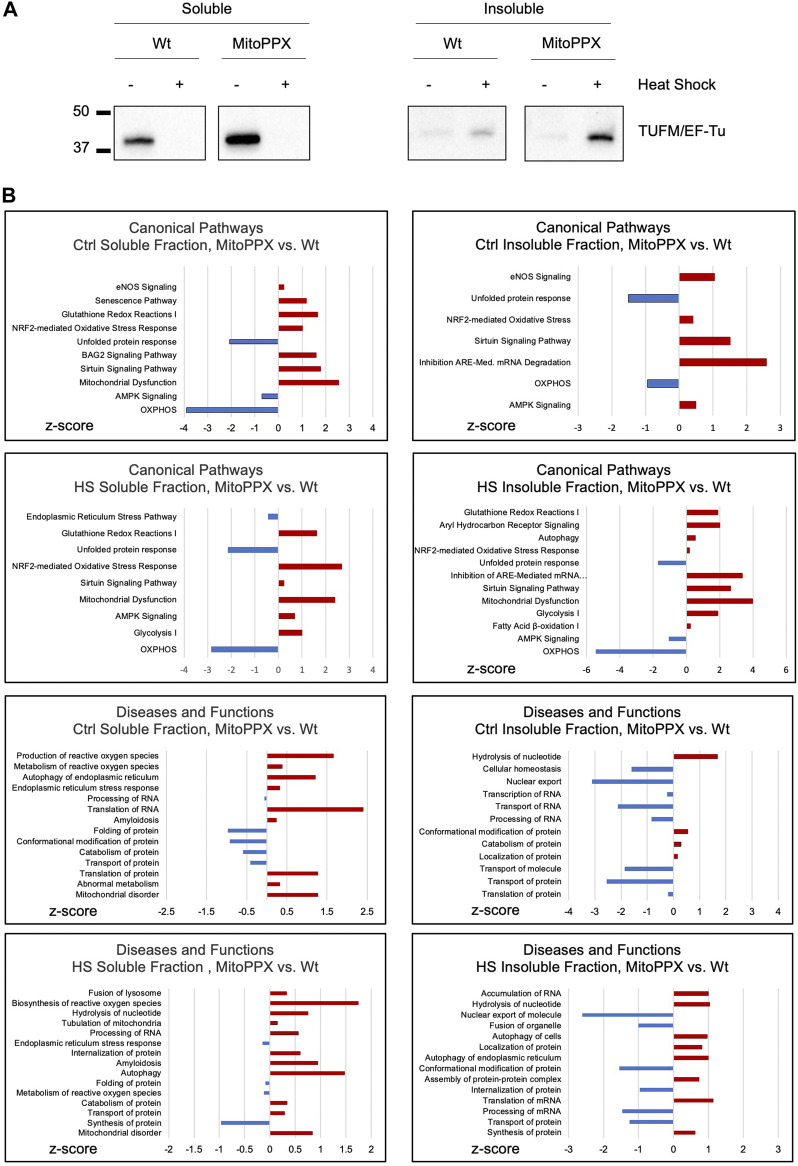
Lack of mitochondrial polyP deleteriously affects protein homeostasis within the organelle. **(A)**. Significant immunoblots showing the aggregation behavior of TUFM under control conditions (25°C) and after HS (30 min, 45°C). Protein content was normalized before separation of the soluble and insoluble mitochondrial fractions by centrifugation. Original uncropped membranes are presented in the Supplementary Material. At least three independent experiments were conducted. **(B).** IPA analysis of the proteomics data obtained from the soluble and insoluble mitochondrial protein fractions of the Wt and MitoPPX HEK293, both under control conditions and after HS. The main canonical pathways; and diseases and functions which are predicted to be affected based on the LC-MS/MS are represented in the graphs. Data is represented as the values obtained in MitoPPX vs. those obtained in Wt cells. Selection of the canonical pathways; and disease and functions were conducted among those with *p*-values ≤0.05, considering z-scores and relationship with mitochondrial physiology and protein homeostasis. Three independent sets of cells were used to obtained the proteomics data. Data sets obtained in the mass spectrometry experiments are included in Supplementary Table S1.

TUFM (mitochondrial translation elongation factor Tu) is the first among the mammalian mitochondrial proteins which aggregates in response to heat stress (Wilkening et al., 2018). Accordingly, we decided to assay the presence of TUFM in the soluble and the insoluble fractions of Wt and MitoPPX cells [cells enzymatically depleted of mitochondrial polyP (Solesio et al., 2016a; Solesio et al., 2020; Hambardikar et al., 2022)], after conducting HS in isolated mitochondria, since it is known that aggregated proteins tend to be insoluble (Hipp et al., 2014; Weids et al., 2016). To separate the soluble and insoluble fractions, equal amounts of mitochondrial protein were used, and samples were centrifugated at high speed. Subsequently, the presence of TUFM was determined in the pellets (insoluble fraction) and supernatants (soluble fraction). Our results show that HS increases the presence of TUFM in the insoluble fraction of MitoPPX cells, when compared with the Wt samples. However, we also detected a higher presence of TUFM in the soluble fraction of MitoPPX mitochondria, compared to Wt, in the absence of HS (Figure 1A).

We then analyzed the proteome of the soluble and insoluble mitochondrial fractions of the Wt and MitoPPX cells, obtained under control conditions and after HS. To do this, we used mass spectrometry (Figure 1B). The results were analyzed using IPA to generate predictions; and the main pathways which were relevant for our studies were selected and plotted based on their z-scores. Regarding the IPA analysis, only pathways with a *p*-value ≤0.05 (−log (*p*-value) higher than 1.3) were included in the analysis. Major differences regarding the affected pathways were found between Wt and MitoPPX cells, both in the soluble and insoluble fractions, and under control and HS conditions. For example, under control conditions, both soluble and insoluble fractions of MitoPPX cells are predicted to show upregulation of cellular stress response, as well as downregulation of OXPHOS and unfolded protein response (UPR), when compared to the Wt cells. Notably, after HS, mitochondrial dysfunction-related pathways are predicted to be upregulated in the soluble and insoluble fractions of the MitoPPX cells. Furthermore, the analysis of the insoluble fraction revealed that MitoPPX cells are predicted to exhibit impaired proteostasis; as evidenced by the upregulation of autophagy, and cellular stress response (canonical pathways); as well as of protein synthesis and transport (disease and functions pathways); and the downregulation of the UPR. The complete proteomics data sets are included in Supplementary Table S1.

### 3.2 Mitochondrial polyP can bind mammalian mitochondrial proteins. The number and the specific type of proteins bound are different under control and stress conditions

**FIGURE 2:**
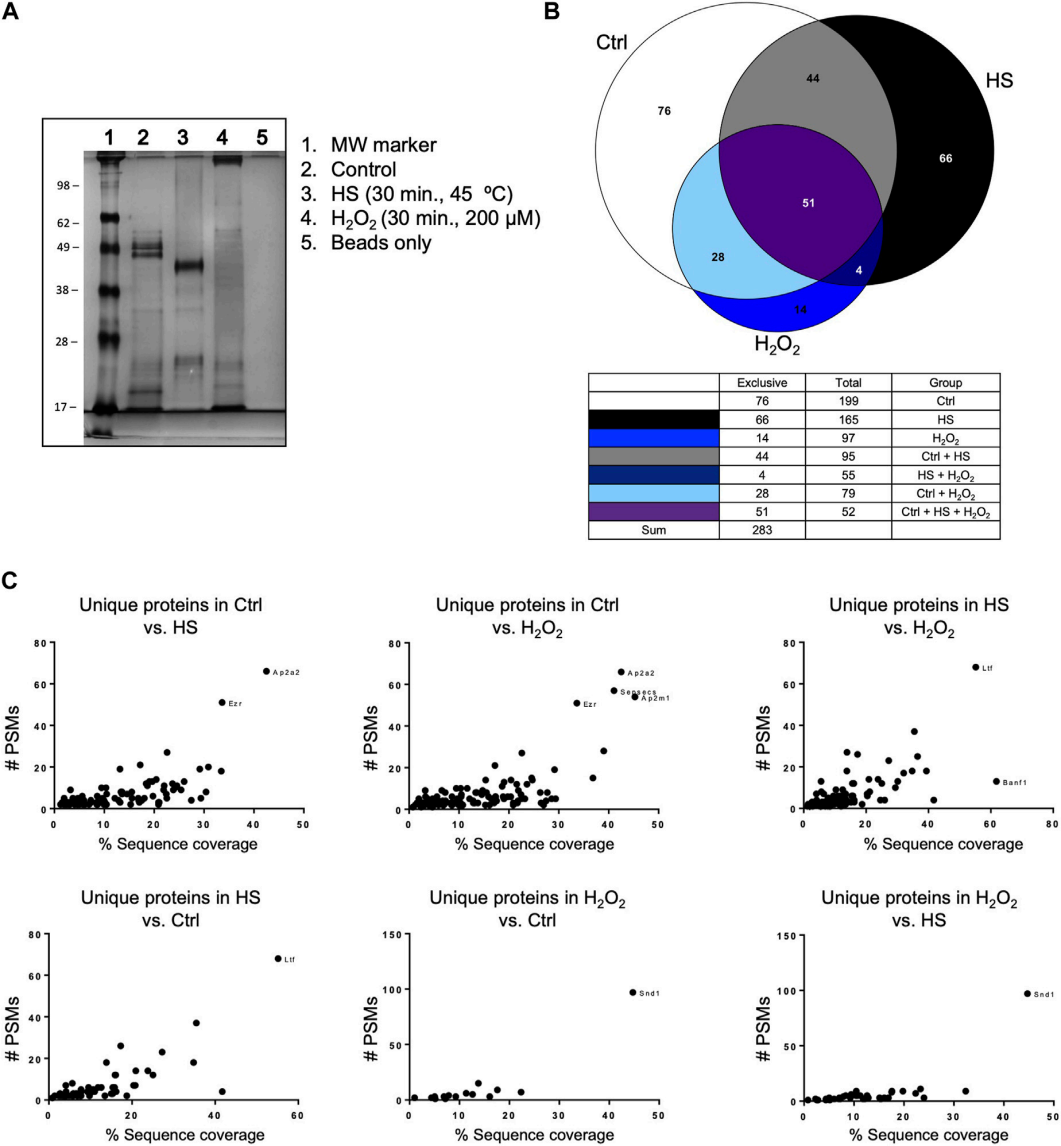
Mitochondrial polyP is able to bind proteins. This binding is affected by the presence of different stressors. **(A)** Coomassie-stained SDS-PAGE showing the crude extracts pulled-down with polyP (lanes 2, 3, and 4). The treatments (HS or H_2_O_2_) and the pull-down were conducted on isolated mitochondria from mice liver. HS was conducted for 30 min at 45°C, and the treatment with H_2_O_2_ was conducted for 30 min, using 200 μM. **(B)** Venn Diagrams showing the number of proteins that were present in each of the studied conditions and the overlapping areas. Proteins were detected by LC-MS/MS **(C)**. Scatter plots showing unique proteins that were present in the comparison between the different conditions. To show this comparison, the results were plotted as PSMs vs. % of sequence coverage. The outliers are the proteins that are more abundant in each of the comparisons, and each dot represents a different protein. Following the standards in the pull-down field, this experiment was conducted only once. The name of these proteins, the exact number of PSMs, and the complete LC-MS/MS results are included in Supplementary Table S2.

We performed a pull-down of proteins bound to polyP, using isolated mitochondria from mice liver. The pull-down experiments were conducted under both control and stress conditions, specifically increased ROS and HS (Figure 2A). Following the analysis of the proteins that were present in each condition using LC-MS/MS, we plotted the data using Venn diagrams (Figure 2B). Our analysis reveals that stress has a potent effect on protein binding to polyP. For example, under control conditions, a total of 199 proteins were pulled-down with polyP, out of which 76 were found to be bound to polyP in this condition. After HS, a decreased number of proteins bound to polyP was observed (165). Among these proteins, 66 were bound to polyP in this condition. Lastly, exposure of mitochondria to H_2_O_2_ resulted in a further decrease of the total number of proteins bound to polyP (97 proteins). In this condition, only 14 proteins were bound to polyP.

Additionally, we plotted the number of peptide spectral matches (PSMs) vs. % coverage data to provide further evidence of significant differences in the studied conditions (Figure 2C). The PSMs analysis complemented the initial findings by identifying and quantifying proteins binding to polyP under different conditions tested. Our findings revealed specific protein interactions with polyP under different stress conditions. Specifically, we observed that lactoferrin Ltf binds to polyP after HS, while protein staphylococcal nuclease-binding domain containing 1 (SND1) showed binding after oxidative stress. The complete mass spectrometry data set is included in Supplementary Table S2, and further experimental details from this assay are included in Supplementary Figure S1.

### 3.3 The enzymatic depletion of mitochondrial polyP affects the expression levels of some of the main genes and proteins which are involved in the maintenance of mammalian protein homeostasis

To further elucidate the mechanisms that explain the differences observed between Wt and MitoPPX cells in the levels of insolubility of TUFM, we assayed the effects of polyP in the regulation of some of the genes that encode proteins that are involved in mammalian protein homeostasis. In this case, to induce stress, we increased the levels of ROS, since the role of these increased levels on activating the stress response has been broadly demonstrated (Schieber and Chandel, 2014). To accomplish this, we used rotenone, a well-known inhibitor of complex I from the electron transfer chain (ETC) (Li et al., 2003). Short-term treatment and subtoxic treatment with rotenone were chosen to avoid any cellular and/or mitochondrial compensatory effects, as well as to decrease the chances of inhibition of protein import within the organelle. In fact, multiple studies show the need for increased length of treatment and/or concentration of rotenone to induce major effects on the ETC in mammalian cells, including HEK293 (Seo et al., 1999; Koopman et al., 2005; Chen et al., 2007; Teixeira et al., 2018). The use of different stressors also contributes to elucidate whether the observed effects in the case of treatment with rotenone are a consequence of differences in mitochondrial protein import. However, we recognize that we cannot completely rule out the possibility that some our observed effects could be a consequence of the inhibition of the ETC by rotenone. To determine the specific conditions of the pharmacological treatment with this drug, we conducted cell viability assays (Supplementary Figure S2).

**FIGURE 3:**
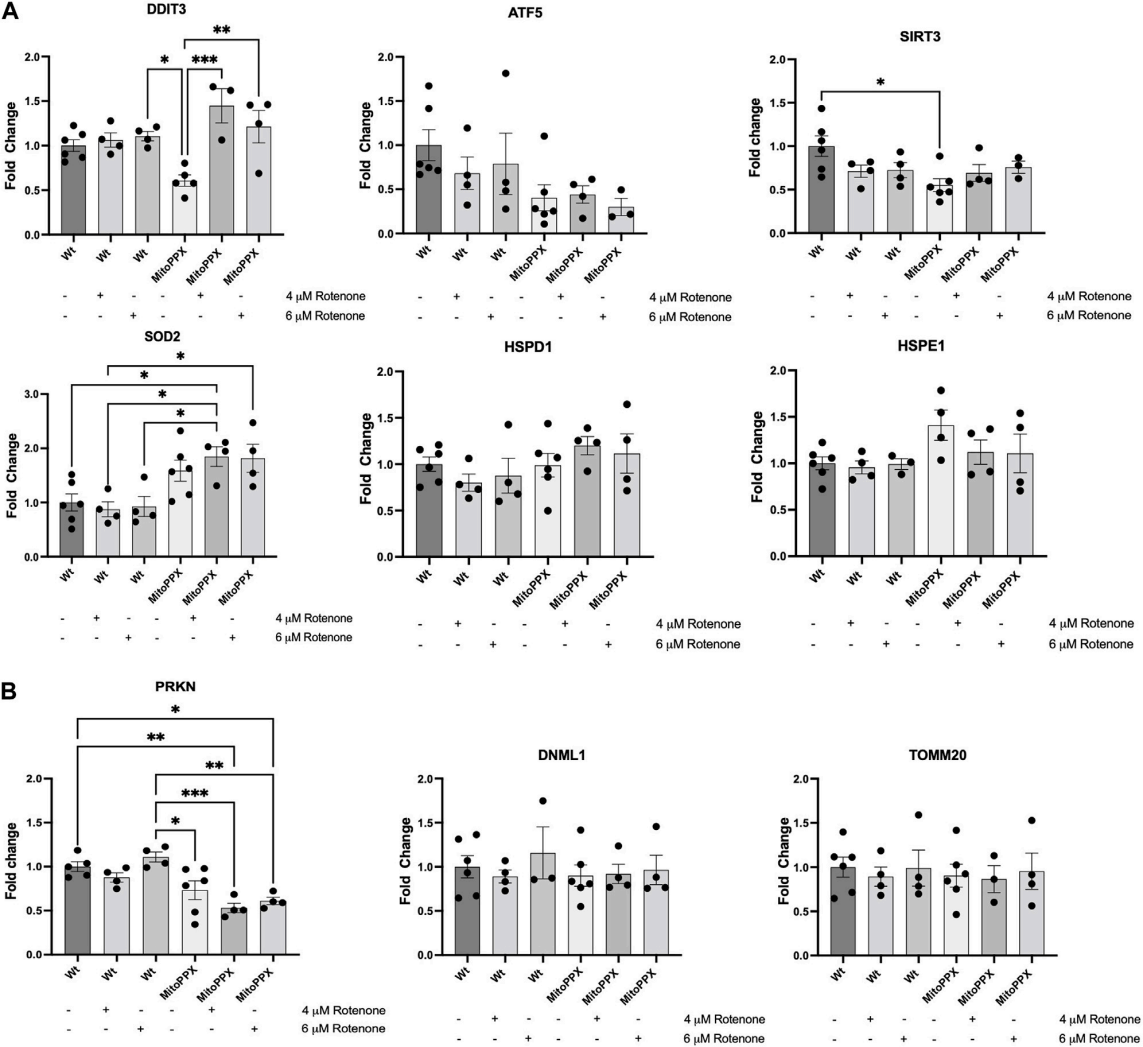
The enzymatic depletion of mitochondrial polyP is a potent regulator of mammalian gene expression. qRT-PCR assays were conducted on samples obtained from at least three independent experiments. Specifically, we assayed the expression levels of some key genes involved on the maintenance of **(A)** Mitochondrial proteostasis (DDIT3, ATF5, SIRT3, SOD2, HSPD1, and HSPE1), and **(B)**. Mitochondrial physiology, further than proteostasis (PRKN, DNML1, and TOMM20) in Wt and MitoPPX HEK293 cells. Experiments were conducted under control conditions and after treatment with rotenone (4 and 6 μM for 3 h). Values were standardized with those found in Wt control. The statistical analysis was carried out by one-way ANOVA with Tukey’s post-test for multiple comparisons; with the exemption of HSPE1, whose statistical analysis was carried out with Brown-Forsythe and Welch ANOVA due to the unequal variances between groups compared. Data is expressed as mean ± SEM from at least three independent experiments. Unless otherwise indicated, the differences between the groups are not significant. *p ≤ 0.05, **p ≤ 0.01, and ***p ≤ 0.001.

Based on the bibliography, we decided to study the levels of gene expression levels of DDIT3 (DNA damage-inducible transcript 3) (Martinus et al., 1996; Zhao et al., 2002), ATF5 (activating transcription factor 5) (Fiorese et al., 2016; Melber and Haynes, 2018), SIRT3 (sirtuin 3) (Papa and Germain, 2014; Kenny et al., 2017), SOD2 (superoxide dismutase 2) (He et al., 2016; Kenny et al., 2017), HSPD1 (heat shock protein family D member 1) (Benedetti et al., 2006; Haynes et al., 2007; Lin et al., 2016), and HSPE1 (heat shock protein family E member 1) (Voos, 2013; Beck et al., 2016) (Figure 3A). The regulation of these genes, and of the proteins encoded by them, have been proposed to be involved in the regulation of the mitochondrial UPR (UPR^mt^) (Munch, 2018), which is a potent mechanism to counteract increased protein dyshomeostasis in the organelle, even if its exact regulation still remains unclear. For example, the interplay between DDIT3 and ATF5 is dynamically regulated during the activation of the UPR (Teske et al., 2013). Accordingly, the expression of DDIT3 and ATF5 were examined as an approach to investigate the early activation (regulated by ATF5 expression) and late activation (regulated by DDIT3 expression) of UPR. Moreover, SIRT3 and SOD2 encode for proteins which are crucial in the regulation of mitochondrial physiology and the antioxidant defense, respectively (Palma et al., 2020; Zhang et al., 2020). Interestingly, SIRT3 can also indirectly regulate the expression and activity of SOD2 (Ong and Logue, 2023). Additionally, HSPD1 and HSPE1 are mitochondrial chaperones that have been associated to transcription factors involved in the activation of the UPR^mt^ (Benedetti et al., 2006; Haynes et al., 2007; Voos, 2013; Beck et al., 2016; Lin et al., 2016). As a housekeeper gene, we used GAPDH (Barber et al., 2005). Our results show a significant increase in DDIT3 expression after treatment with rotenone (4 μM and 6 μM), in MitoPPX cells, compared to the same cells under control conditions. We also show a constitutive decreased expression of SIRT3, and upregulated SOD2 in MitoPPX after treatment with rotenone, in both cases when compared with the Wt samples. No significant differences in the expression of ATF5, HSPD1, and HSPE1 were detected.

Using the same methods, we also assayed the expression levels of some of the main genes involved in the regulation of mitochondrial physiology [PRKN, and DNML1; they are crucial regulators of mitochondrial proteostasis (Thorne and Tumbarello, 2022)]; as well as of TOMM20, to determine whether protein import was impacted by treatment with rotenone (Figure 3B). Our results show a clear decreased expression of PRKN in MitoPPX cells, specifically after the treatment with rotenone, when compared with the Wt samples, while no differences are present in the expression levels of TOMM20 and DNML1.

**FIGURE 4:**
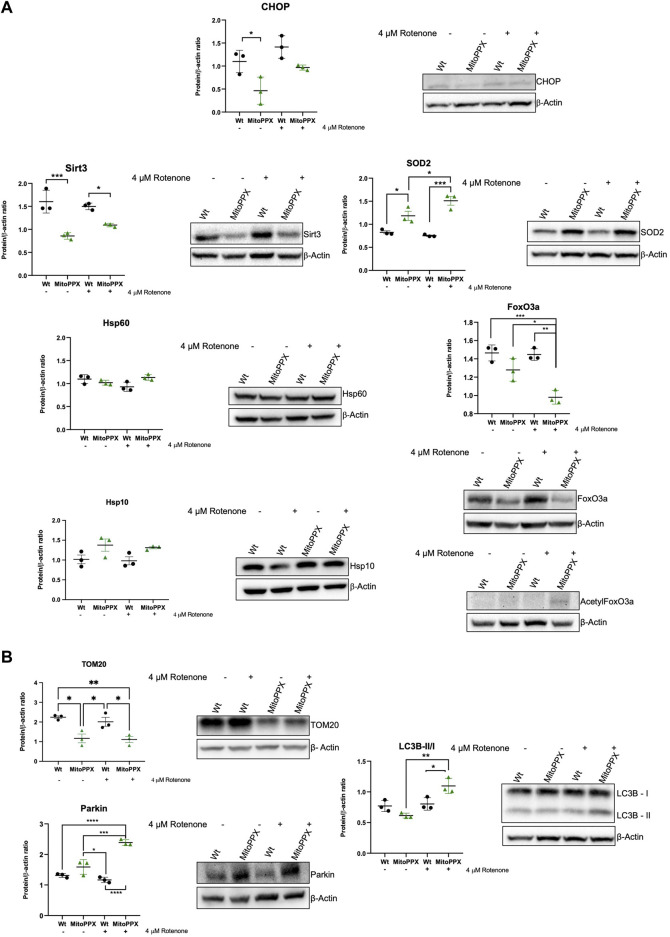
Proteins involved in the maintenance of mitochondrial proteostasis are affected by the depletion of mitochondrial polyP. Representative immunoblots analysis and quantification of protein levels obtained from cell lysates. In this case, we followed the same rationale as for the studies of gene expression, and we assayed some key proteins involved in the maintenance of **(A)**. Mitochondrial proteostasis (CHOP, Sirt3, SOD2, Hsp60, Hsp10, and FoxO3a), and **(B)**. Mitochondrial physiology, further than proteostasis (TOM20, Parkin, and LC3 I/II). To conduct these experiments, Wt and MitoPPX cells were used under control conditions and after treatment with rotenone 4 μM for 3 h to increase protein dyshomeostasis via a rise in the generation of mitochondrial ROS. Original uncropped membranes are included in the Supplementary Material. Data is expressed as mean ± SEM from at least three independent experiments. One-way ANOVA followed by Tukey’s multiple comparisons test was performed to determine statistical significance. Otherwise indicated, the differences between the groups are not significant. *p ≤ 0.05, **p ≤ 0.01, ***p ≤ 0.001, and ****p ≤ 0.0001.

Subsequently, we assayed the levels of some of the main proteins which have been described to be involved in the maintenance of mammalian protein homeostasis. Following the same rationale as in our studies of gene expression, we assayed CHOP (DDIT3 gene), Sirt3, SOD2, Hsp60 (HSPD1), and Hsp10 (HSPE1). We also assayed the levels of FoxO3a (forkhead box O3a) and acetylated FoxO3a, which is another protein usually involved in the maintenance of mitochondrial proteostasis, whose effects are tightly controlled by post-translational modifications (Mouchiroud et al., 2013). In this case, we did not analyze ATF5 due to the lack of reliable commercial antibodies for this protein. Our immunoblots corroborate some of the data obtained by PCR (Figure 4A). For example, under control conditions, CHOP expression is decreased in MitoPPX compared to Wt cells. Additionally, decreased Sirt3 is observed in MitoPPX cells, even if this effect seems to be more drastic when we assayed protein presence over gene expression. There is also a clear increase in the levels of SOD2 in MitoPPX cells, compared to the Wt samples, especially after the treatment with rotenone. Finally, no significant changes in Hsp60 and Hsp10 were observed between Wt and MitoPPX cells, which corroborates our findings obtained by PCR analysis. In the case of FoxO3a, the levels of the protein are clearly decreased in MitoPPX cells after treatment with rotenone, when compared with the Wt samples. However, acetylation is of a higher magnitude in the case of the MitoPPX cells, especially after treatment with rotenone (Figure 4A).

Lastly, also here, we assayed the levels of some of the main proteins involved in the maintenance of mitochondrial physiology (Figure 4B). Our data show that the depletion of mitochondrial polyP affects the mitochondrial outer membrane import system, as demonstrated by changes in the levels of TOM20. Moreover, contrarily to what we describe in the PCR data, the protein levels of Parkin are increased in MitoPPX cells after treatment with rotenone. Furthermore, we assayed the LC3B-II/I ration, since mitochondrial and cellular proteostasis seem to be connected with autophagy (Aman et al., 2021); and LC3B is a well-described marker of autophagy (Tanida et al., 2008). LC3B is also one of the main components of the cellular UPR, which is mostly orchestrated by the ER (Luhr et al., 2019). Our data show an activation of this pathway in MitoPPX cells after treatment with rotenone.

## 4 Discussion

The pivotal regulatory role of polyP in mammalian bioenergetics has been extensively demonstrated by our research and others (Solesio et al., 2016a; Solesio et al., 2020; Borden et al., 2021; McIntyre and Solesio, 2021; Solesio et al., 2021; Scoma et al., 2023). Furthermore, the intricate interplay between bioenergetic status and proteostasis, along with the involvement of polyP in preserving bacterial proteostasis and regulating amyloidogenesis, are known (Gray et al., 2014; Nargund et al., 2015; Cremers et al., 2016; Yoo et al., 2018; Lempart et al., 2019; Lempart and Jakob, 2019; Xie and Jakob, 2019). However, little is known regarding the role of polyP on the maintenance of mitochondrial protein homeostasis. In this study, we delve into this role for the first time, shedding light on its significance. To disturb proteostasis in our samples, we used increased ROS and HS, which are well-known stressors in mammalian cells (Duncan and Hershey, 1984; Mizzen and Welch, 1988; Abe et al., 1999; Squier, 2001; Kim et al., 2011; Levy et al., 2019). We used both insults to show that our results are not dependent on the specific type of stimuli.

Our data show the chaperoning effect of polyP against the aggregation of mitochondrial proteins. Specifically, we studied the distribution of TUFM between the soluble and insoluble fractions of mitochondria to conduct these studies. We chose TUFM because it has been demonstrated that this is the first mitochondrial protein to aggregate under stress conditions (Wilkening et al., 2018). We found an increased presence of TUFM in the insoluble fraction of MitoPPX cells, when compared with Wt samples. However, the presence of TUFM was already increased in the soluble fraction of the MitoPPX cells under control conditions. One plausible explanation for these observed differences could be that the depletion of mitochondrial polyP in MitoPPX cells leads to unbalanced energy homeostasis, as we have already shown in our previous studies (Solesio et al., 2021; Hambardikar et al., 2023). Unbalanced bioenergetics could ultimately compromise the efficiency of protein folding and assembly within mitochondria. Furthermore, the overexpression of TUFM in the soluble fraction of MitoPPX cells could be a compensatory response to the mitochondrial stress induced by the depletion of mitochondrial polyP. Specifically, upregulated TUFM could be an attempt to enhance mitochondrial protein translation, ultimately mitigating impaired proteostasis. However, after HS, high levels of TUFM accumulates in the insoluble fraction. Moreover, MitoPPX HEK293 cells do not present impaired cell viability, or any major structural changes, as we have previously shown (Solesio et al., 2016a; Solesio et al., 2020; Solesio et al., 2021; Hambardikar et al., 2022). Additionally, the role of polyP as a bacterial chaperone is already known (Dahl et al., 2015; Cremers et al., 2016; Yoo et al., 2018), and this mechanism could be conserved in mammalian cells, especially considering how well polyP is conserved throughout evolution. Considering all this, the increased levels of TUFM in the soluble fraction of the MitoPPX cells could be interpreted as a protective mechanism to mitigate the deleterious effects caused by the depletion of polyP on the chaperoning ability of the organelle.

To further study whether the depletion of mitochondrial polyP affects protein homeostasis within the organelle, we analyzed the proteome of the soluble and insoluble mitochondrial fractions from Wt and MitoPPX cells, under control and HS conditions. Our IPA predictions show that HS induces significant changes in the proteins that are present in the different mitochondrial fractions of MitoPPX cells. For example, OXPHOS is clearly downregulated in MitoPPX cells when compared to Wt samples, even under control conditions and in both fractions. A considerable number of the protein complexes involved in OXPHOS are encoded in mitochondrial DNA. Therefore, increased proteotoxicity due to decreased levels of mitochondrial polyP could contribute to the dysfunction of OXPHOS, which could further contribute to increased proteotoxicity (due to the high demands of energy of chaperones), creating add deleterious feedback. Our data also show upregulated UPR and mitochondrial dysfunction in both the soluble and insoluble fraction of MitoPPX cells, after HS. The upregulation of these pathways is already predicted in MitoPPX cells under control conditions. These findings suggest that the potent effects of polyP on mitochondrial protein homeostasis are already exerted under basal conditions, and they are more dramatic when proteotoxic stress is present. Additionally, upregulated autophagy is present in the insoluble fraction of MitoPPX cells after HS, which suggest that the activation of the stress response when polyP is depleted in mitochondria is of a bigger scope.

The pull-down of proteins bound to polyP revealed substantial changes in under control and stress (increased ROS and HS) conditions. For example, our findings show that HS induces a higher number of proteins to be pulled-down compared to increased ROS. This suggests that HS induces a more pronounced interaction between polyP and proteins. Moreover, we found that Ltf binds to polyP in HS but not under control conditions. Ltf is a glycoprotein with various effects in mammalian cells, including binding to organic iron to protect against oxidative stress (Kowalczyk et al., 2022). The finding that Ltf binds to polyP under HS conditions suggests that it may play a role in the response against protein dyshomeostasis, potentially contributing to the cellular defense mechanisms during stress. Furthermore, after treatment with H_2_O_2,_ we found that SND1, also known as P100, is bound to polyP. This binding is not present under control conditions. SND1 is a well-conserved protein among eukaryotes. It is found in mitochondria, where it plays multifaceted roles. Recently, mitochondrial SND1 has been reported to increase under stress conditions. In fact, it has been demonstrated that SND1 is recruited to mitochondria in response to stress stimulus and that it promotes mitophagy through the PGAM5—Drp1^S635^ dephosphorylation pathway (Liang et al., 2022). Accordingly, our findings align with the existing literature, which shows that polyP can bind to mammalian proteins (Morrissey et al., 2012), and highlights the potent role of the polyP in maintaining protein homeostasis in bacteria and in mammalian amyloidogenesis (Gray et al., 2014; Dahl et al., 2015; Yoo et al., 2018; Lempart et al., 2019; Lempart and Jakob, 2019; Xie and Jakob, 2019). They also suggest a significant regulatory role for polyP in mammalian protein homeostasis and provide insights into the cellular mechanisms involved in the mammalian stress response and adaptation.

HS and oxidative stress are distinct cellular stressors that can elicit diverse responses in biological systems, including mitochondria (Slimen et al., 2014). This is further confirmed by our pull-down data. Therefore, we decided to address whether the protective effects of polyP on mitochondrial proteostasis are conserved independently of the specific stressor. Since the studies with TUFM were conducted using HS, we decided to address the status of gene expression and protein levels in response to sub-toxic concentrations of rotenone.

The enzymatic depletion of mitochondrial polyP induces a sharp effect in the expression of some of the main genes in charge of maintaining mitochondrial proteostasis, including some of those proposed to be involved in the UPR^mt^, both under control conditions and in response to treatment with rotenone. For example, our data show increased expression of DDIT3 in MitoPPX cells in response to rotenone, which suggest the late activation of the UPR (Ong and Logue, 2023), especially considering the lack of differences in the expression of ATF5. In fact, previous studies have shown that the upregulation of DDIT3 mRNA is associated with a prolonged ER stress pathway (Tsai et al., 2017; Yu et al., 2022). The activation of the UPR and the UPR^mt^ are intimately related in mammalian physiology, and the number of publications showing a close relationship between the physiology of mitochondria and ER has increased in the last decade (Zhang et al., 2023). Therefore, by dysregulating mitochondrial proteostasis, the depletion of polyP within the organelle could have a global effect in the cell. As mentioned above, the expression of ATF5 do not show substantial changes between our different experimental conditions. While ATF5 has been described as one of the main regulators of the mammalian UPR^mt^, current knowledge suggests that the effects of ATF5 in mitochondrial protein homeostasis are mediated by the mitochondrial levels of the protein, and not necessarily by its expression levels (Fiorese et al., 2016). Moreover, other regulatory mechanisms of the UPR^mt^, independent of ATF5, may also be present in mammalian cells.

Our data also show that, under control conditions, the enzymatic depletion of mitochondrial polyP induces a constitutive downregulation in the expression of SIRT3, a protein involved in the maintenance of mitochondrial physiology, including protein homeostasis (Papa and Germain, 2014; Zhang et al., 2020). In fact, the downregulation of SIRT3 can disrupt the protein quality control mechanisms within mitochondria, including the UPR^mt^ (Meng et al., 2019), and potentially trigger cellular stress responses. However, when stress is present, the levels of SIRT3 are similar in Wt and MitoPPX cells, which suggest the existence of a compensatory mechanism. Our data also show increased expression of SOD2 in the MitoPPX cells. SOD2 is one of the main mitochondrial antioxidant enzymes in mammalian cells (Flynn and Melov, 2013). We have already demonstrated that even if MitoPPX HEK293 cells are viable and fully functional, they have increased levels of ROS, probably due to dysregulated OXPHOS (Solesio et al., 2021; Hambardikar et al., 2022). Probably as a compensatory effect, an increased presence of antioxidants in MitoPPX cells has been reported (Hambardikar et al., 2022). Therefore, the rise in the expression of SOD2 could be related to the dsyregulation of bioenergetics, instead of being directly linked to mitochondrial proteostasis. Furthermore, we did not find significant differences in the expression of HSPD1 and HSPE1 after rotenone treatment. These two genes encode for two of the main mammalian chaperones: Hsp60 and its co-chaperone Hsp10, respectively. The lack of regulatory effects of polyP in these genes corroborates the results obtained in the ATF5 assay, since ATF5 is a major regulator of mitochondrial chaperones, including Hsp60 and Hsp10 (Fiorese et al., 2016). Accordingly, our data suggests that the protective effects of polyP on proteostasis are not mediated by the regulation of the expression of the classical mitochondrial chaperones. Moreover, our results show that PRKN gene expression is decreased in MitoPPX cells, especially under treatment conditions. This effect is not observed when we assayed the protein levels. Lastly, no changes in the expression levels of DNM1L and TOMM20 were observed, which suggest that the effects of polyP on these cells are circumscribed to the regulation of protein homeostasis.

Immunoblots corroborate much of our previous regarding gene expression findings. However, we found two exceptions to this: TOM20 and Parkin. Increased Parkin-mediated mitophagy is associated with degradation of mitochondrial membranes, and therefore it can lead decreased levels of TOM20, which are found in our studies (Yoshii et al., 2011). An increased presence of Parkin in the MitoPPX cells also suggests a rise in the activation of mitophagy. In this case, the differences between the gene expression levels and the protein presence could be explained by the multiple post-translational modifications that Parkin undergoes to be active, including phosphorylation (Durcan and Fon, 2015). Depletion of mitochondrial polyP could modify phosphate homeostasis in mitochondria. Moreover, polyP is able to polyphosphorylate proteins (Azevedo et al., 2015; Azevedo et al., 2018; Bentley-DeSousa et al., 2018). All this could affect the levels of active Parkin. Decreased levels of TOM20 could further affect mitochondrial protein homeostasis. TOM20 is a translocase of the outer mitochondrial membrane, which is part of the TOM (transporter outer membrane) protein import machinery. TOM has been demonstrated to be impaired in human disease, including neurodegeneration (Chai et al., 2018; Franco-Iborra et al., 2018). In fact, dysregulated mitochondrial protein import could trigger proteotoxic stress in both the organelle and the cytosol. Furthermore, deficits in mitochondrial protein import could affect the import of proteins required for proper mitochondrial functioning, including some of the components of the ETC. Therefore, decreased levels of polyP could also indirectly affect mitochondrial proteostasis.

To further understand the effects of polyP in cellular physiology, we addressed whether cellular autophagy, a process which is closely related to mitophagy (Glick et al., 2010; Youle and Narendra, 2011), is affected by the depletion of mitochondrial polyP. Specifically, we assayed variations in the LC3B-I/LC3B-II ratio, as this is one of the most common markers of autophagy (Tanida et al., 2008; Bjorkoy et al., 2009). Our results align with those found in Parkin, indicating a rise in the autophagic processes in MitoPPX cells, and corroborate previous findings from both our research group and others, highlighting the global impact of the depletion of mitochondrial polyP on cellular processes. (Bondy-Chorney et al., 2020; Solesio et al., 2021; Hambardikar et al., 2022). Using immunoblots, we were not able to assay the levels of ATF5 due to the lack of reliable antibodies. However, we included FoxO3a (and its acetylated form) in our study. FoxO3a is involved in the regulation of oxygen metabolism and mitochondrial gene expression (Ferber et al., 2012), two processes in which polyP is involved, and which are closely related to the status of mitochondrial proteostasis. While the levels of FoxO3a are decreased in MitoPPX cells when rotenone is present, its acetylation is clearly increased in the same samples. Interestingly, post-translational modifications of FoxO3a modulate the subcellular location of the protein, as well as its activity (Boccitto and Kalb, 2011). For example, in response to stress and after these post-translational modifications, FoxO3a migrates to nuclei and mitochondria, where it is active (Fasano et al., 2019). Moreover, increased acetylation of FoxO3a has been shown to regulate mitophagy besides the antioxidant response (Gupta et al., 2022). Previous studies have demonstrated the close relationship between FoxO3a and Sirt3. Specifically, in response to stress stimuli, Sirt3, which is primarily located in the mitochondria, can deacetylate FoxO3a, likely to protect the organelle against increased oxidative stress by activating the expression of antioxidant enzymes, such as SOD2 (Tseng et al., 2013). Furthermore, deacetylation of FoxO3a upregulates the expression of a set of genes that are involved in the maintenance of mitochondrial proteostasis (Tseng et al., 2013). MitoPPX cells exhibit decreased levels of Sirt3, which correlates with the increased levels of acetylated FoxO3a observed in the same samples. We recognize that the regulation of FoxO3a is complex, and other explanations (such as variations in the antioxidant system) could also underlie our findings.

Some of these findings may suggest that the regulatory influence of polyP on mitochondrial proteostasis operates through the modulation of the transcriptome, rather than directly impacting protein stability. This hypothesis finds support in the known regulatory function of long-chain polyP in the mammalian transcriptome (Bondy-Chorney et al., 2020). However, previous studies examining the protective effects of polyP on protein homeostasis, particularly those focused on elucidating its role in amyloidogenesis, indicate a direct impact of polyP on protein stability (Cremers et al., 2016; Yoo et al., 2018; Lempart et al., 2019). In fact, polyP has already been described as a “polyanionic protein scaffold” (Xie and Jakob, 2019; Guan and Jakob, 2024).

Altogether, our findings show for the first time the critical role played by polyP in the regulation of mitochondrial proteostasis, and they could pave the road for further studies using the metabolism of mitochondrial polyP as a potent pharmacological target in pathologies where mitochondrial protein dyshomeostasis has been demonstrated. These diseases range from neurodegeneration to cancer (Boland et al., 2013; Patro et al., 2021).

## Data Availability

The mass spectrometric data presented in this study are deposited at https://massive.ucsd.edu accession numbers Massive MSV000095168, and MSV000095178.
