# Supplementary material for: Mitochondrial inorganic polyphosphate is required to maintain proteostasis within the organelle
Source: Front Cell Dev Biol. 2024 Jul 10;12:1423208. doi: 10.3389/fcell.2024.1423208 (PMC11266304; doi:10.3389/fcell.2024.1423208)

Supplementary Figure 1

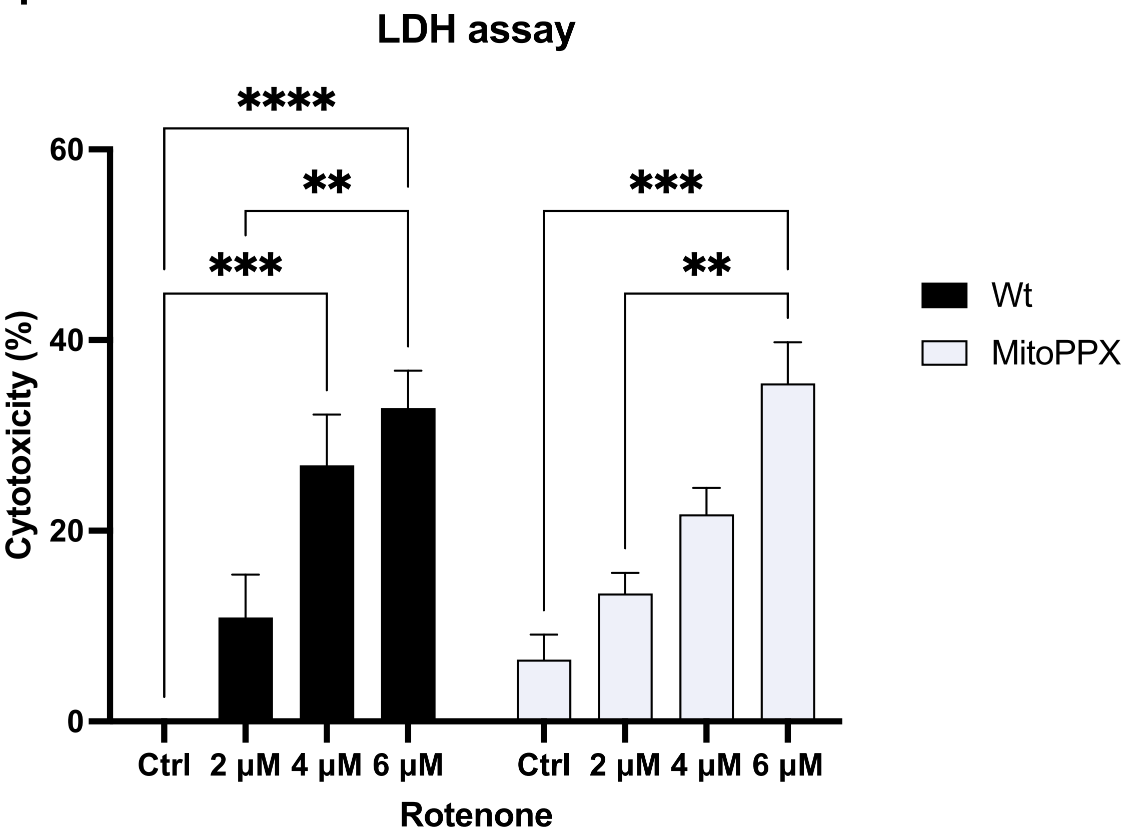

Supplementary Figure 1. Rotenone affects cytotoxicity differently in Wt and MitoPPX cells. LDH assay measuring cytotoxicity in Wt and MitoPPX HEK293 cells in response to increasing concentrations of rotenone during 3 h treatments. The experiment was conducted on at least three independent days and at least triplicates from each experimental condition. All the data were standardized by Wt control values which were set as 0 in each experiment. Statistical differences were detected by two-way ANOVA followed by Tukey's post-test for multiple comparisons. Data is expressed as mean ± SEM. $^{**}p \leq 0.01$, $^{***}p \leq 0.001$ and $^{****}p \leq 0.0001$

Supplementary Figure 2

A

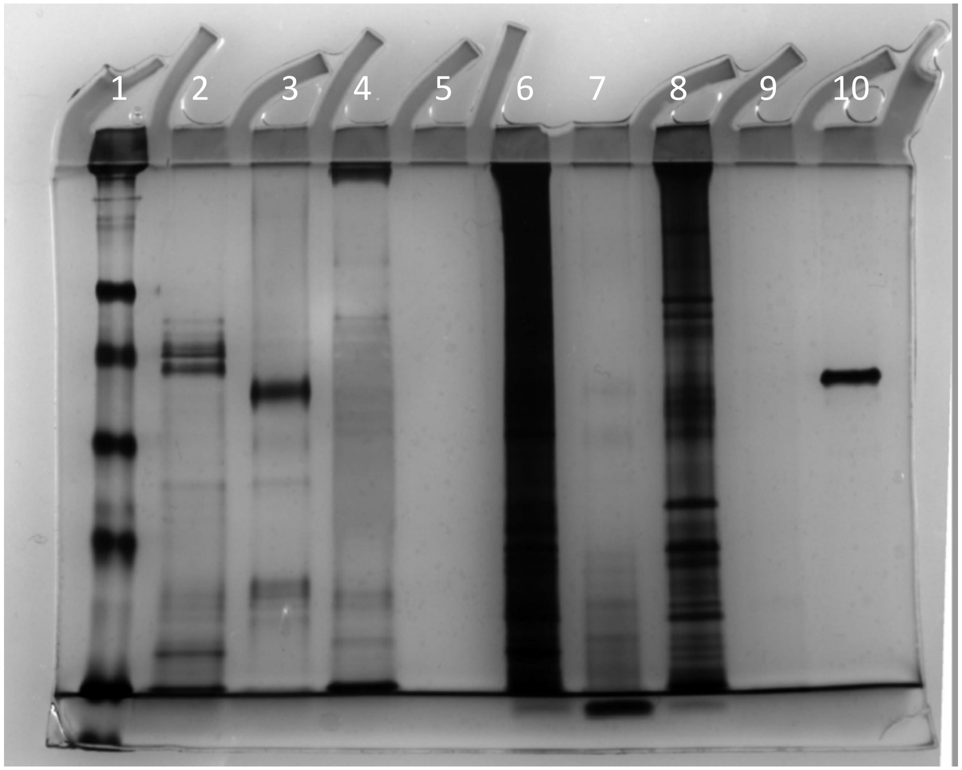

1. Molecular weight marker
2. Control mitochondria
3. HS mitochondria
4. H_2O_2 mitochondria
5. Beads only
6. Control mitochondria (SDS/heat elution)
7. HS mitochondria (SDS/heat elution)
8. H_2O_2 mitochondria (SDS/heat elution)
9. HS + beads only (negative control - SDS/heat elution)
10. 50 ng BSA

B

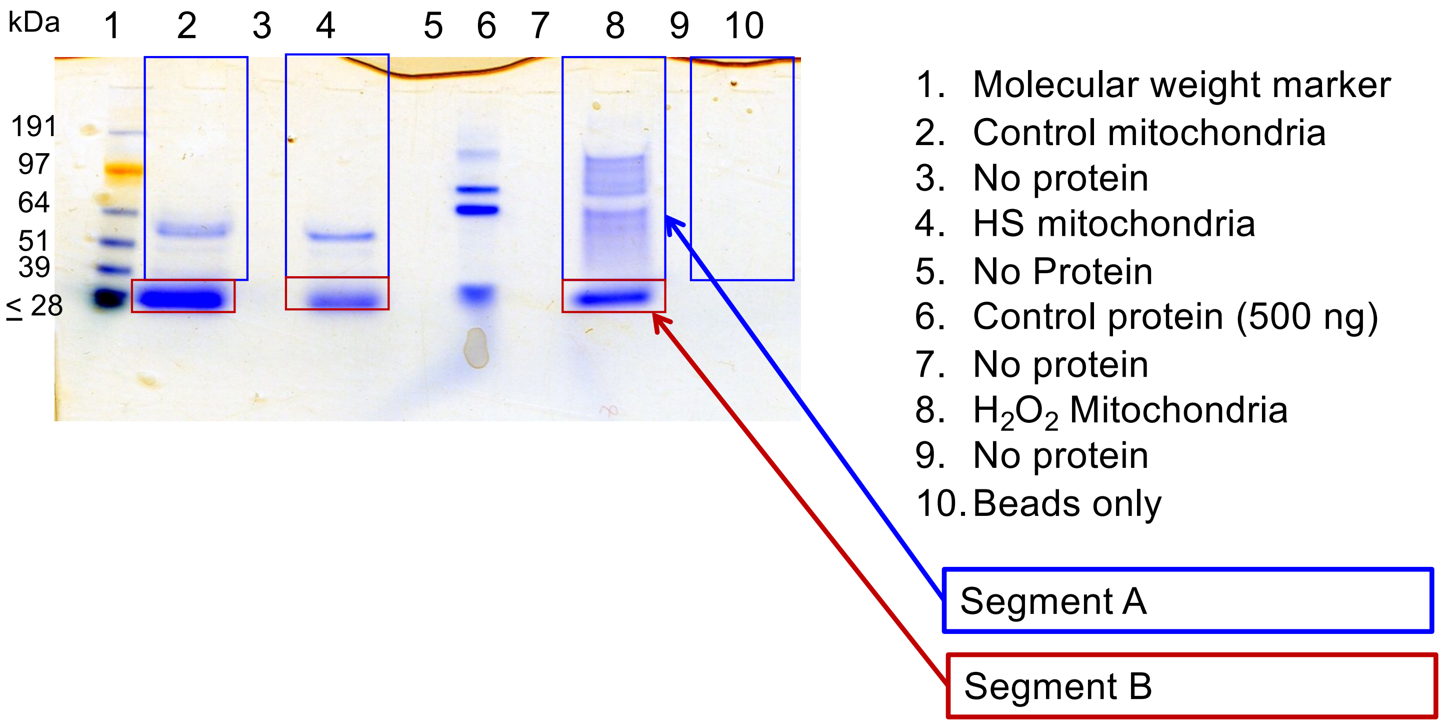
kDa
1 2 3 4 5 6 7 8 9 10
191
97
64
51
39
≤ 28
1. Molecular weight marker
2. Control mitochondria
3. No protein
4. HS mitochondria
5. No Protein
6. Control protein (500 ng)
7. No protein
8. H_2O_2 Mitochondria
9. No protein
10. Beads only
Segment A
Segment B

C BasePeak Chromatograms

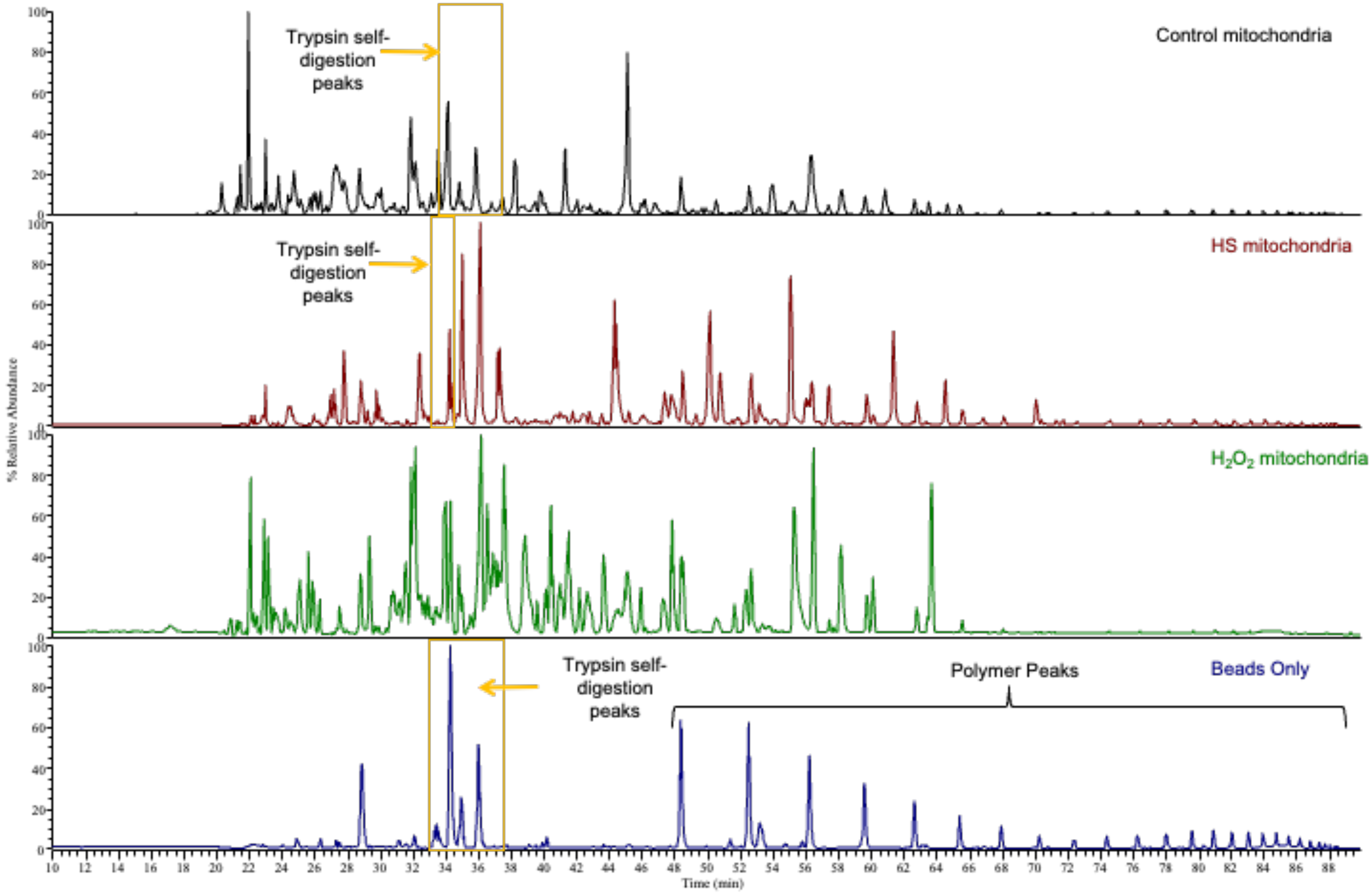

FIGURE LEGEND

Supplementary Figure 2. Supporting data and experimental details regarding the pull-down assay. A. Uncropped gel (10% LiCl elution) showing all the conditions that were assayed, including the controls. **B.** After being reduced and alkylated, samples were run on a NuPAGE 4-12% Bis-Tris Gel. The gel was stained using Coomassie, and the bands used for mass spectrometry were obtained from this gel. The figure shows the segments that were cut to conduct the mass spectrometry assay. The upper and the lower bands (segments A and B, respectively) were cut and prepared separately. Aliquots of the prepared segments A and B for each sample were combined for the LC-MS/MS analysis. Specifically, in the case of control mitochondria, 1/5 of segment A and 1/100 of segment B were added; in the case of HS mitochondria, 1/5 of segment A and 1/50 of segment were added; in the case of H_2O_2 mitochondria, 1/20 of segment A and 1/20 of segment B were added; and in the case of the beads only condition, 1/5 of segment A and no material from the segment B were added. **C.** Base peak chromatograms for each of the samples. The most abundant peptide ions eluting at a given time in each of the conditions, which were detected by the mass spectrometer are plotted. Note that based on these plots, the composition of the samples was substantially different and as expected, the beads only condition had a very limited presence of protein.

Supplementary Table 1. Complete datasheet of LC-MS/MS data corresponding to the experiments conducted in the soluble and insoluble protein fractions of Wt and MitoPPX mitochondria. Mitochondria were isolated after the treatments. Protein fractioning was conducted, and soluble and insoluble fractions were separated prior to mass spectrometry analysis. Values and comparations are included.

Supplementary Table 2. Complete datasheet of LC-MS/MS data corresponding to the experiments conducted in proteins pull-down by polyP. Pull-down and treatments were conducted on isolated mitochondria. Comparisons between the different conditions are included in the different sheets of the excel document.

Uncropped Gels from Figure 3A

Soluble

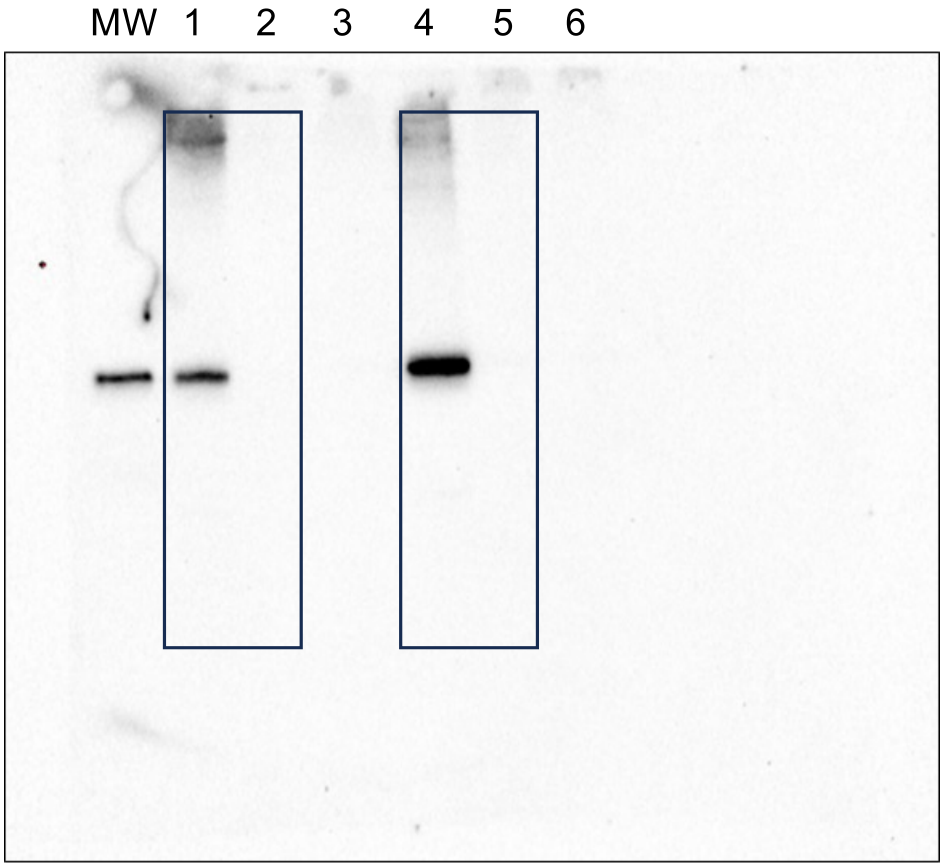

1 Mitochondria HEK293 Wt Ctrl
2 Mitochondria HEK293 Wt HS
3 Other condition
4 Mitochondria HEK293 MitoPPX Ctrl
5 Mitochondria HEK293 MitoPPX HS
6 Other condition

Soluble

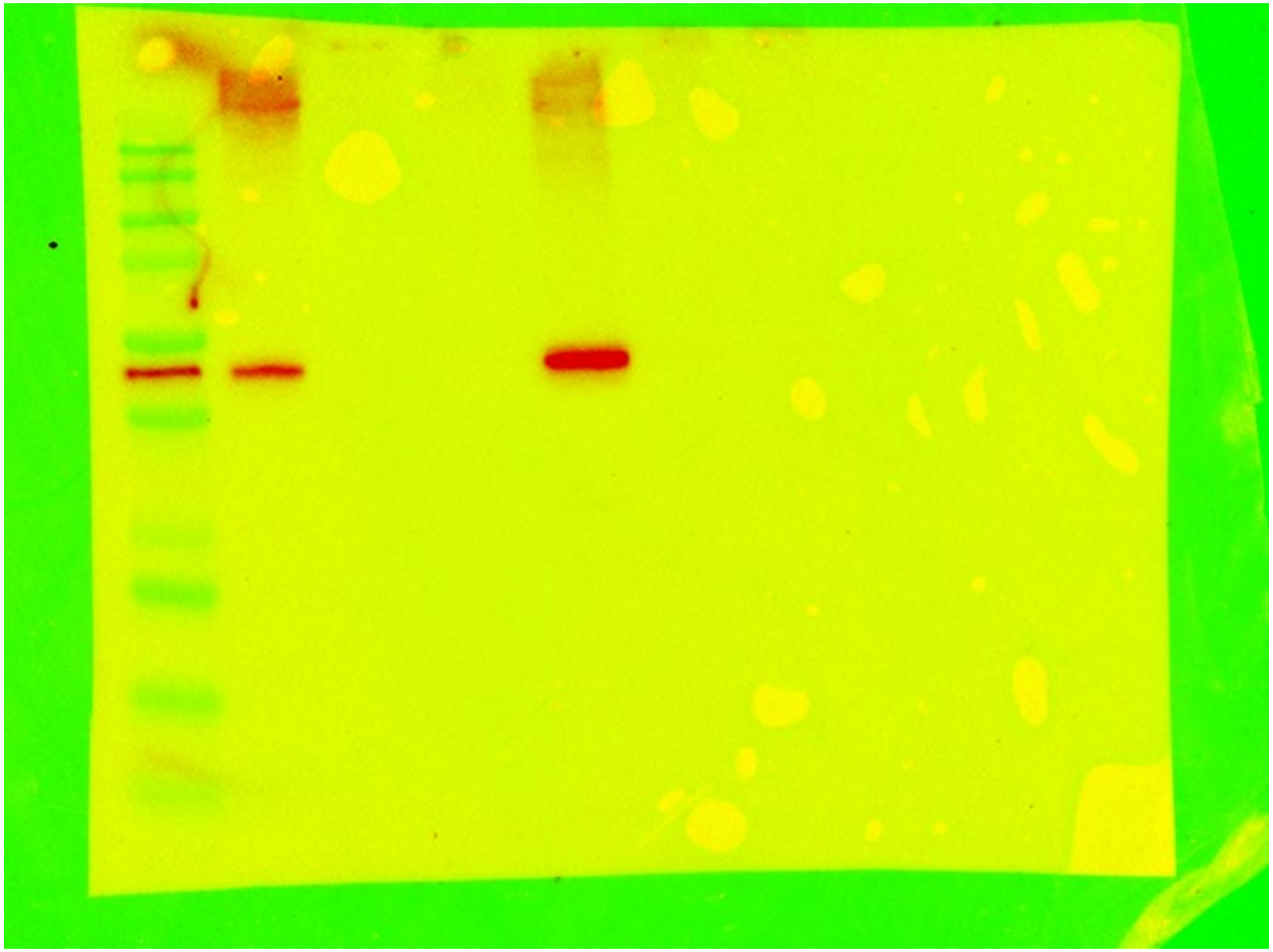

Insoluble

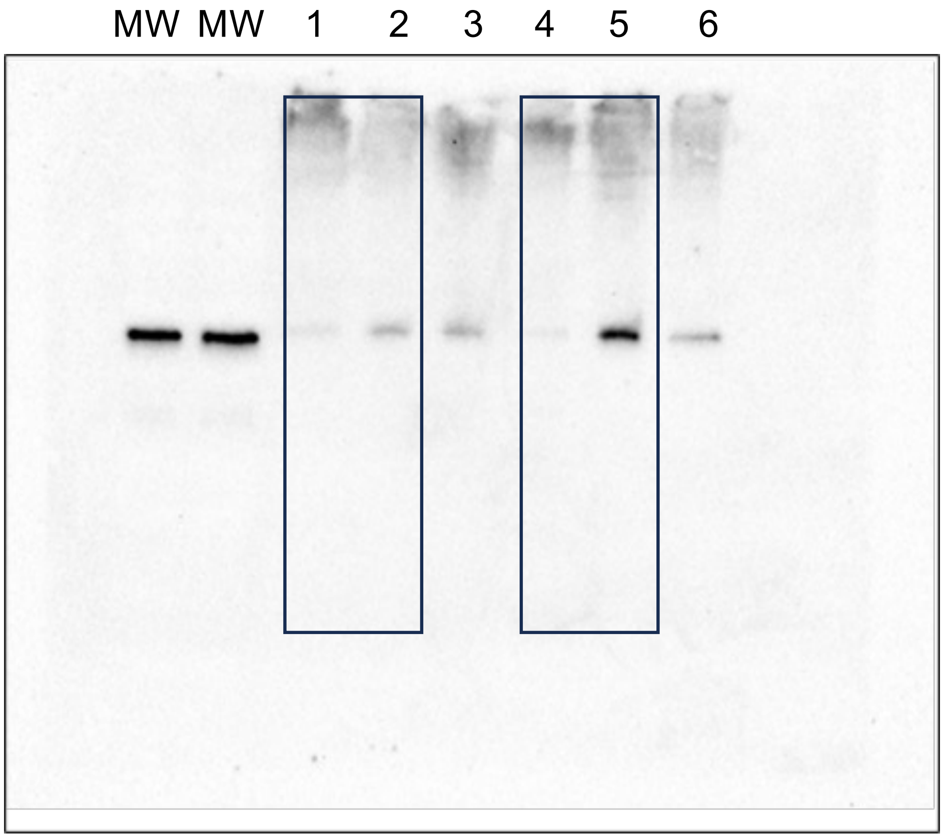

1 Mitochondria HEK293 Wt Ctrl
2 Mitochondria HEK293 Wt HS
3 Other condition
4 Mitochondria HEK293 MitoPPX Ctrl
5 Mitochondria HEK293 MitoPPX HS
6 Other condition

Insoluble

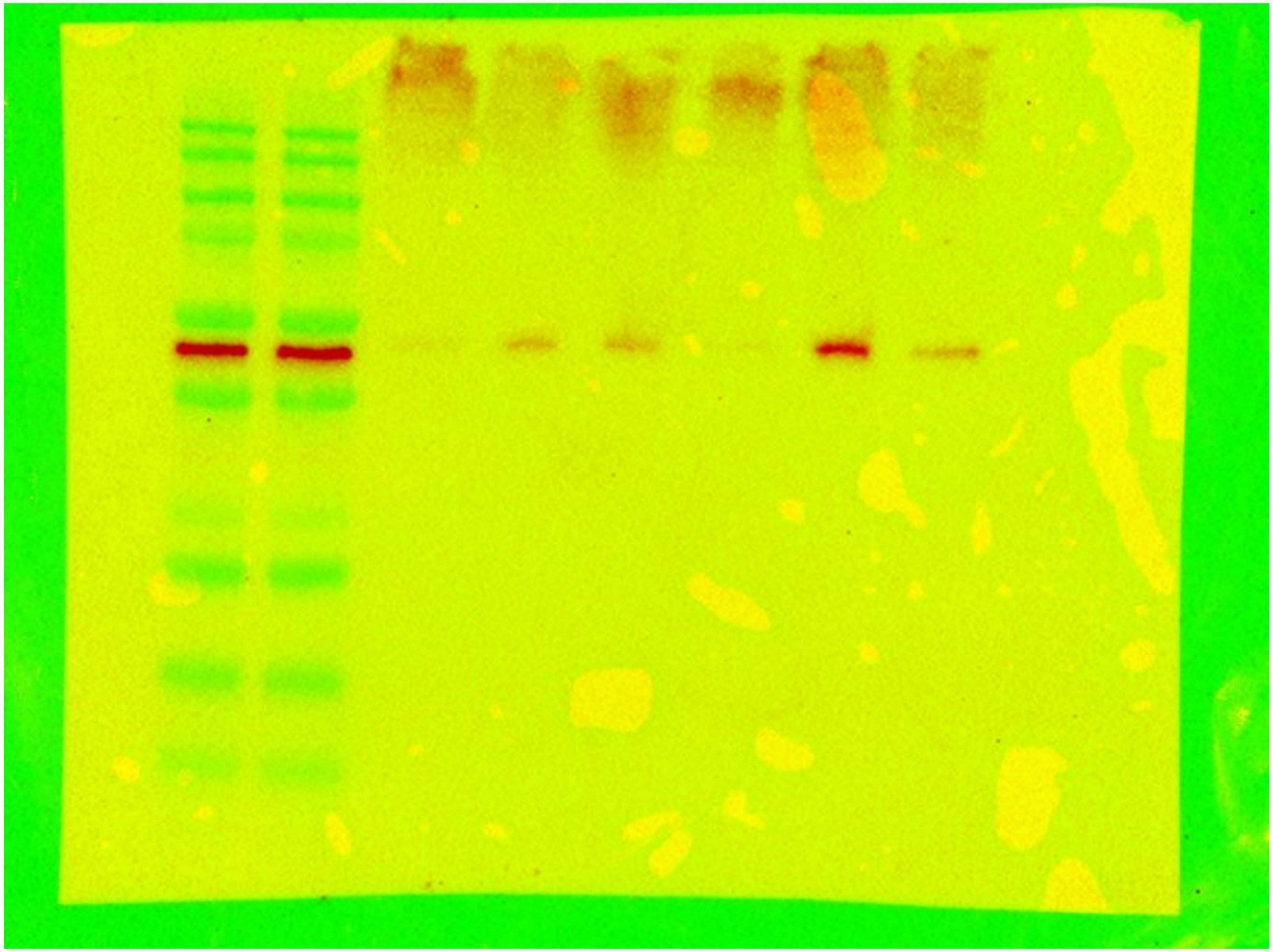

Uncropped Gels from Figure 5A

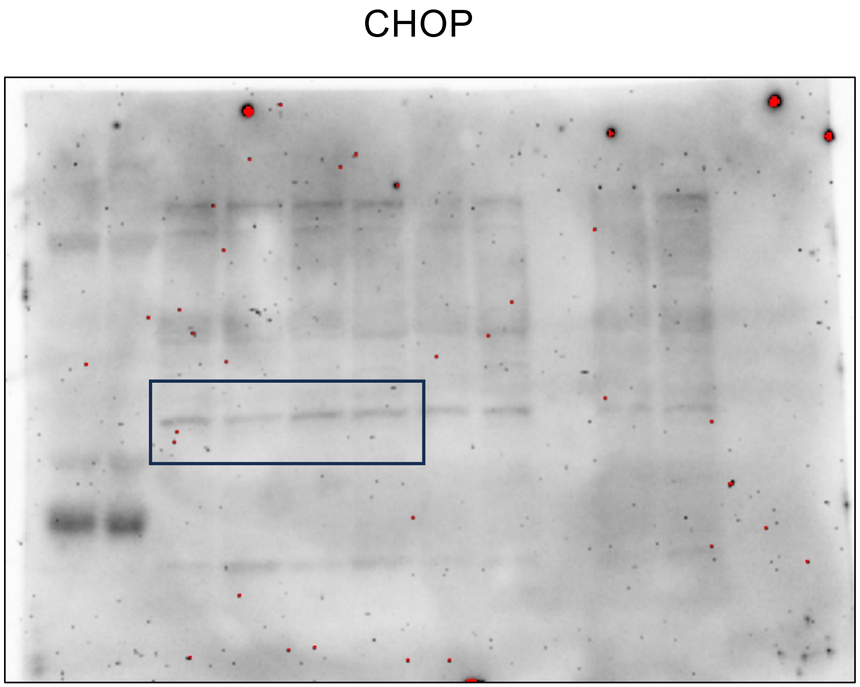

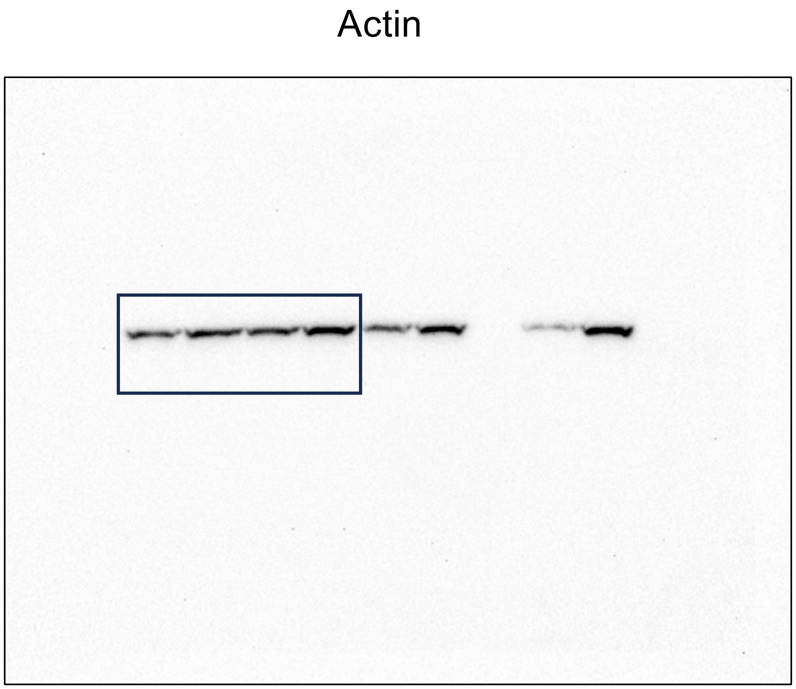

CHOP

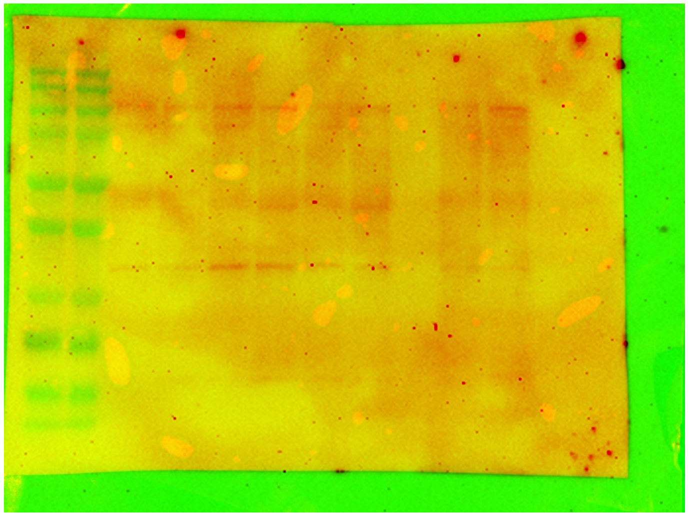

Actin

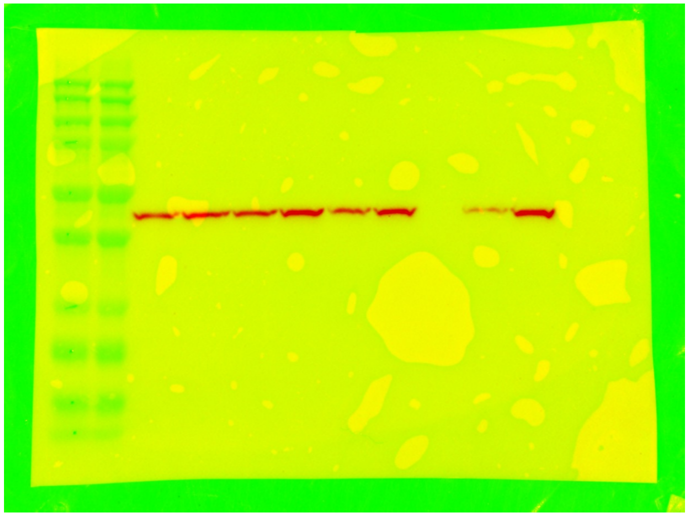

Sirt 3

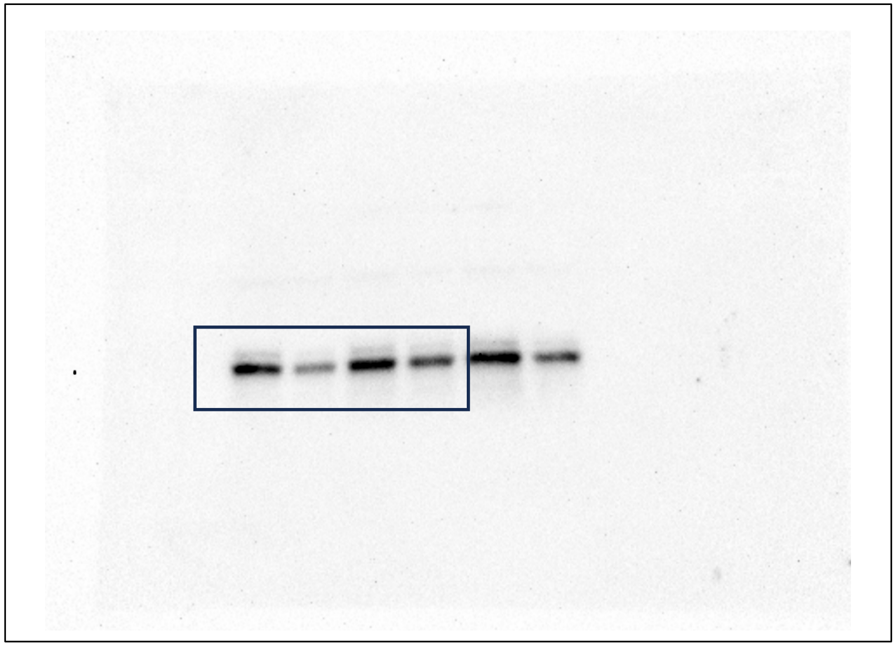

Actin

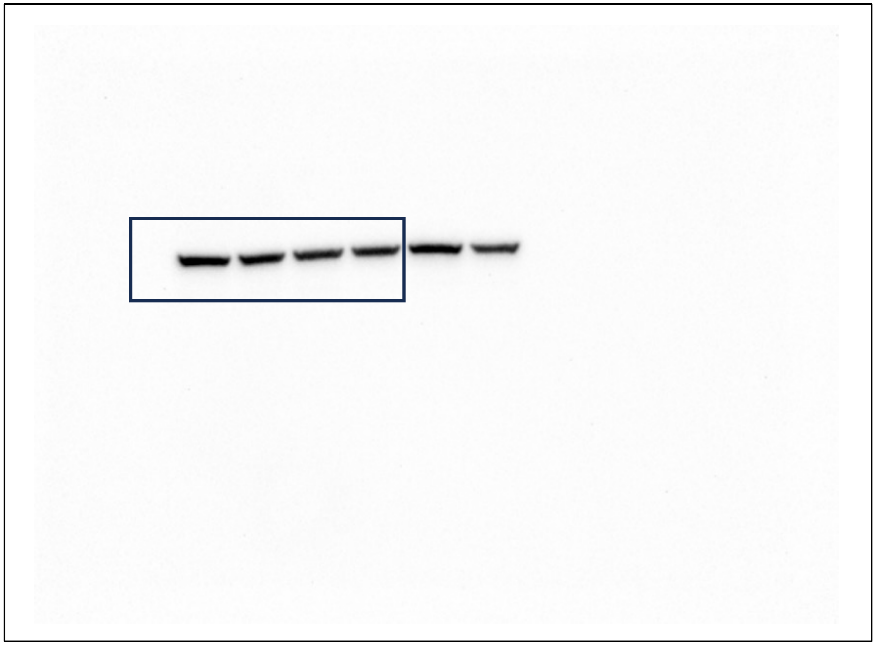

Sirt 3

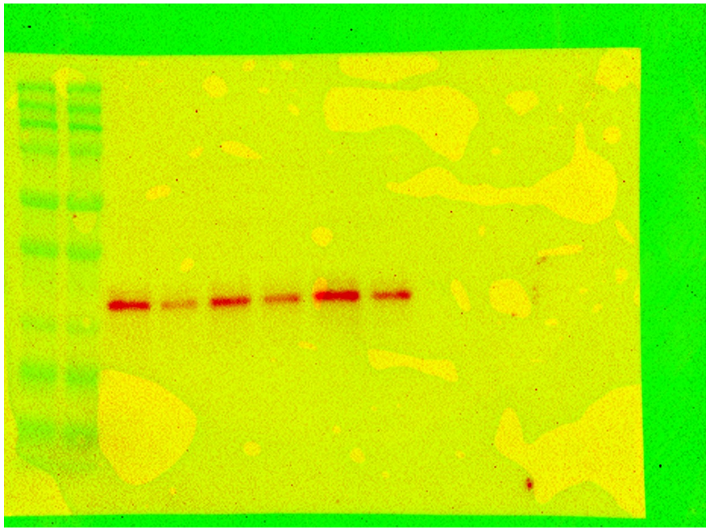

Actin

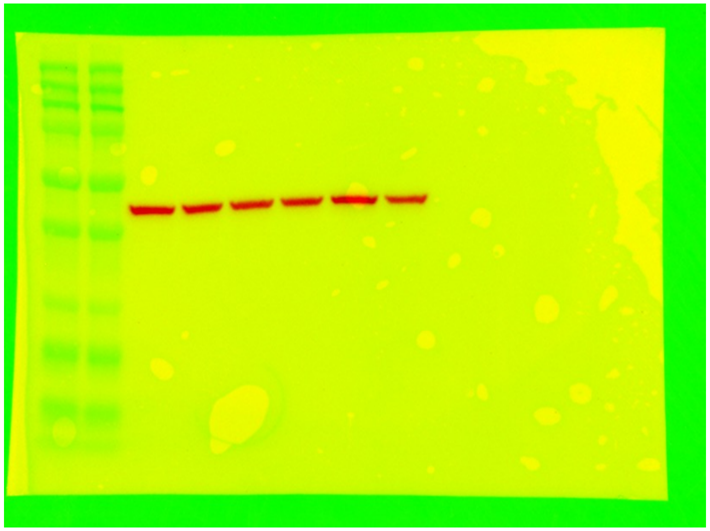

SOD2

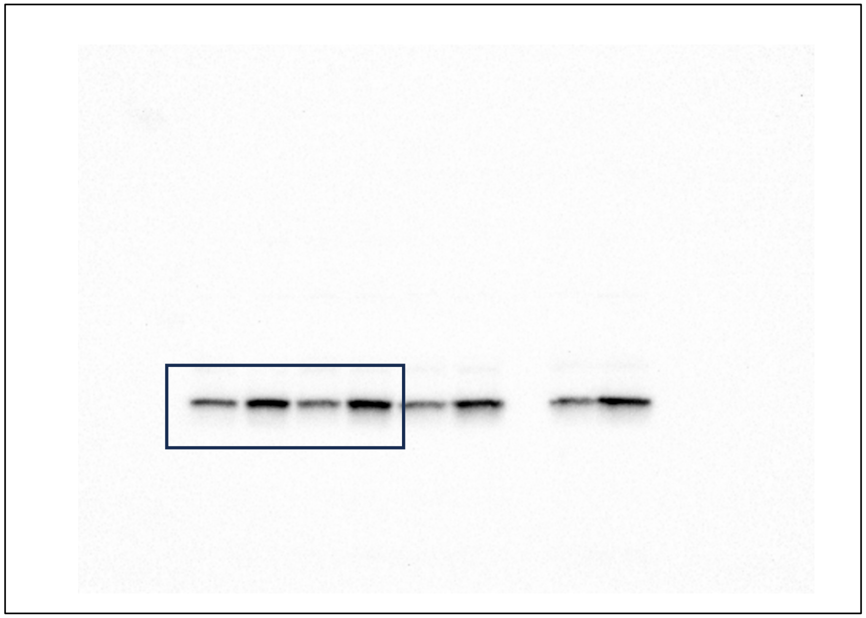

Actin

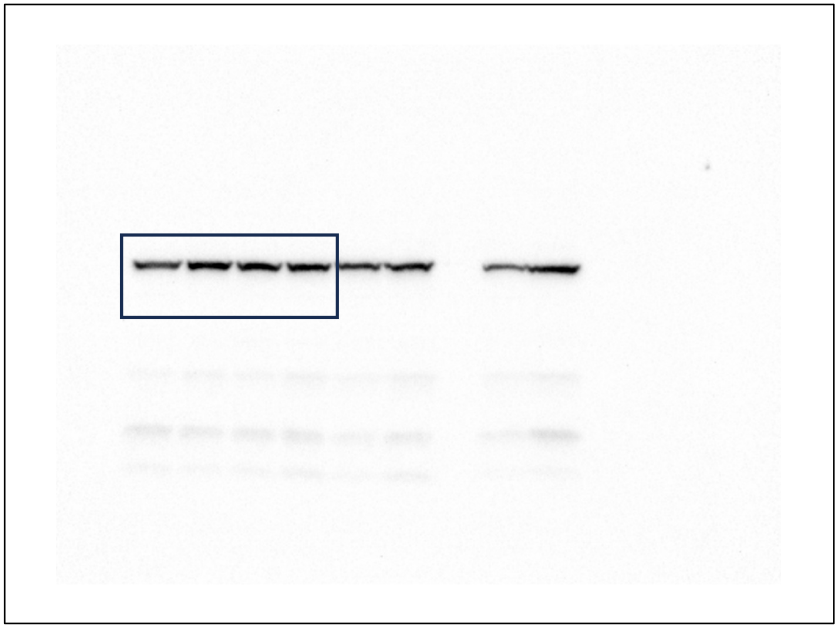

SOD2

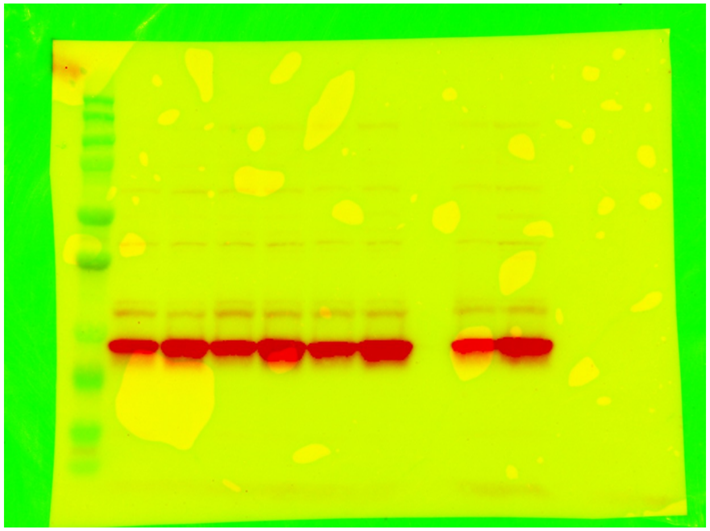

Actin

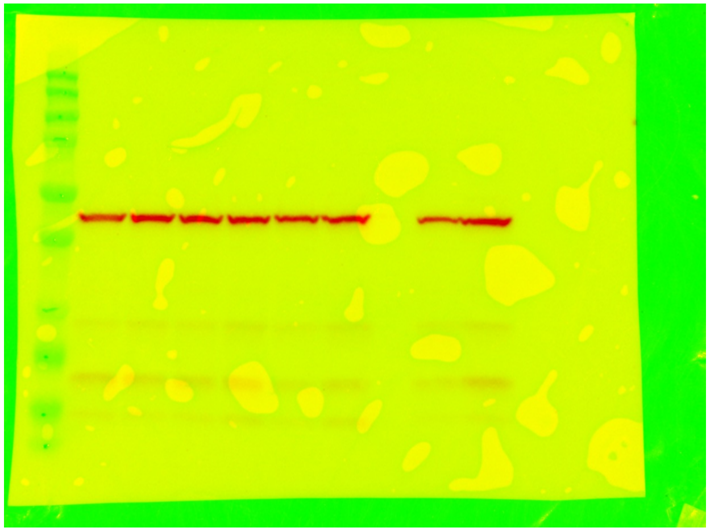

Hsp60

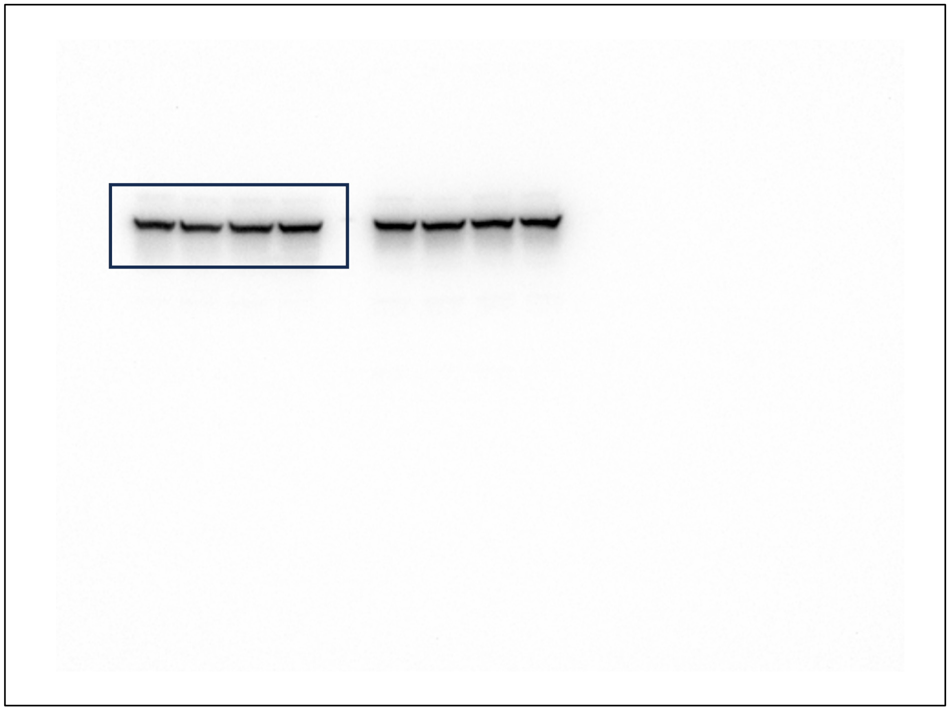

Actin

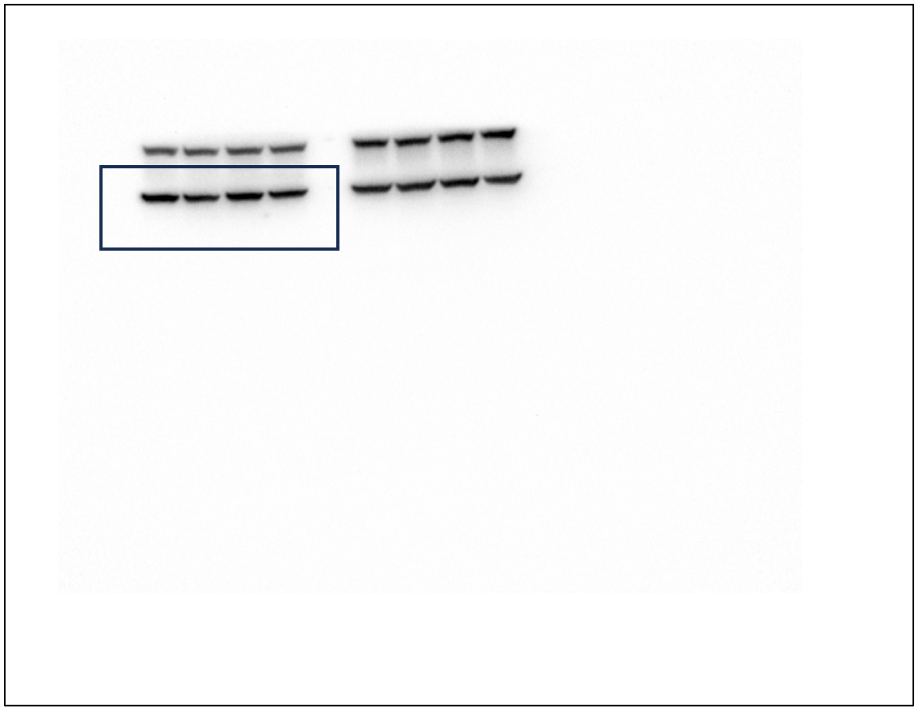

Hsp60

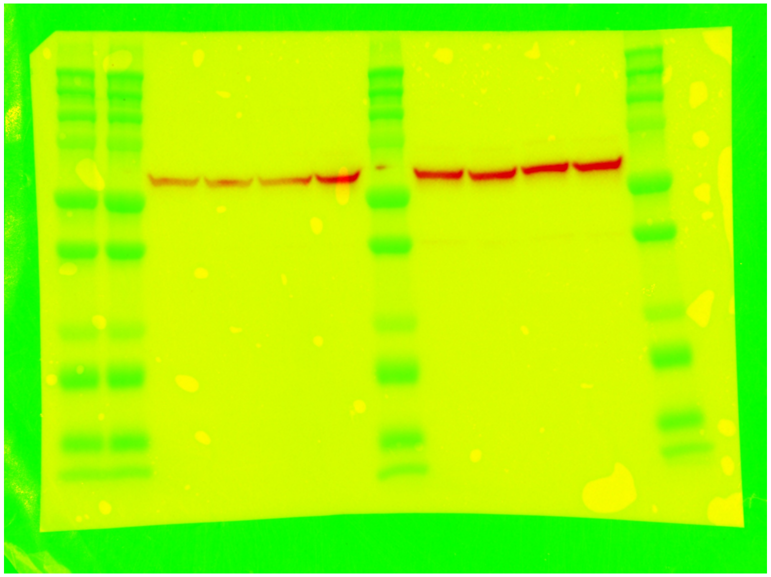

Actin

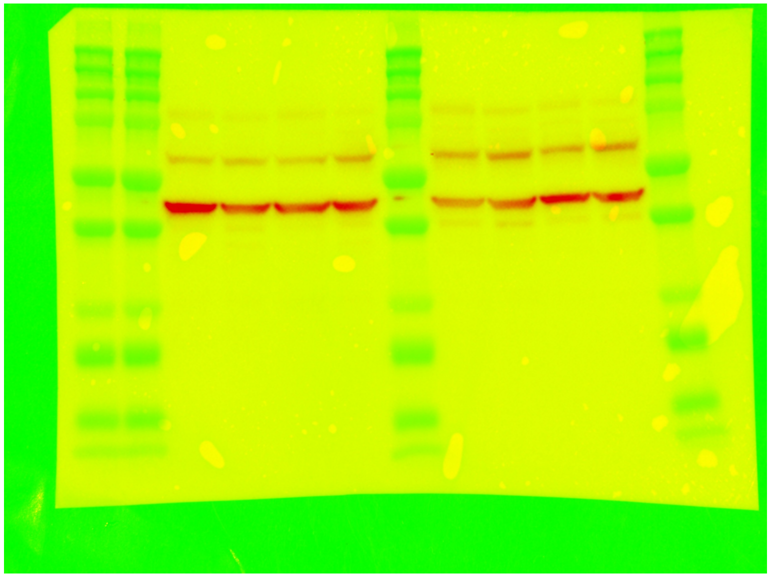

Hsp10

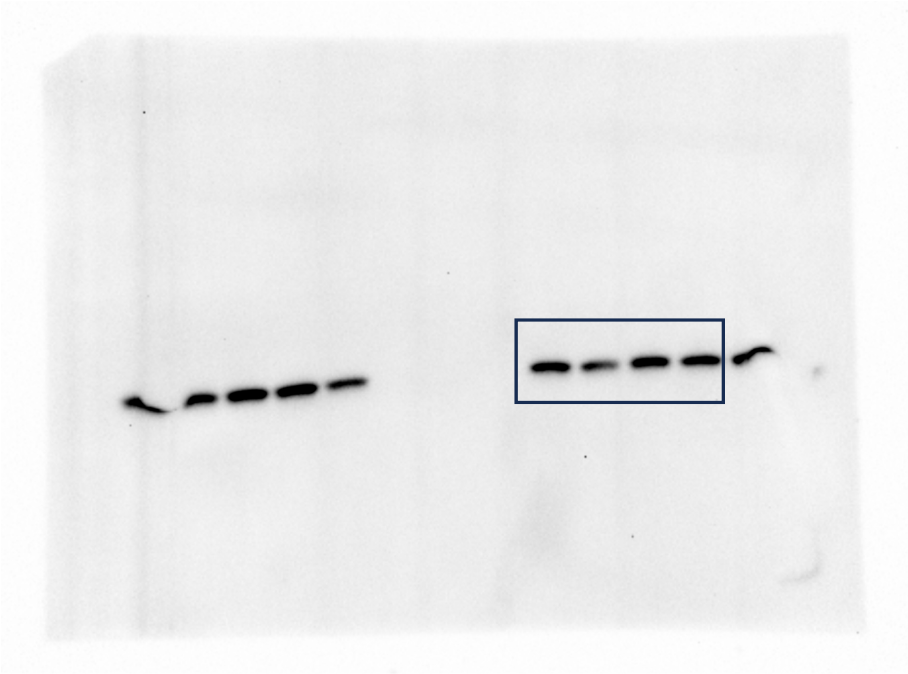

Actin

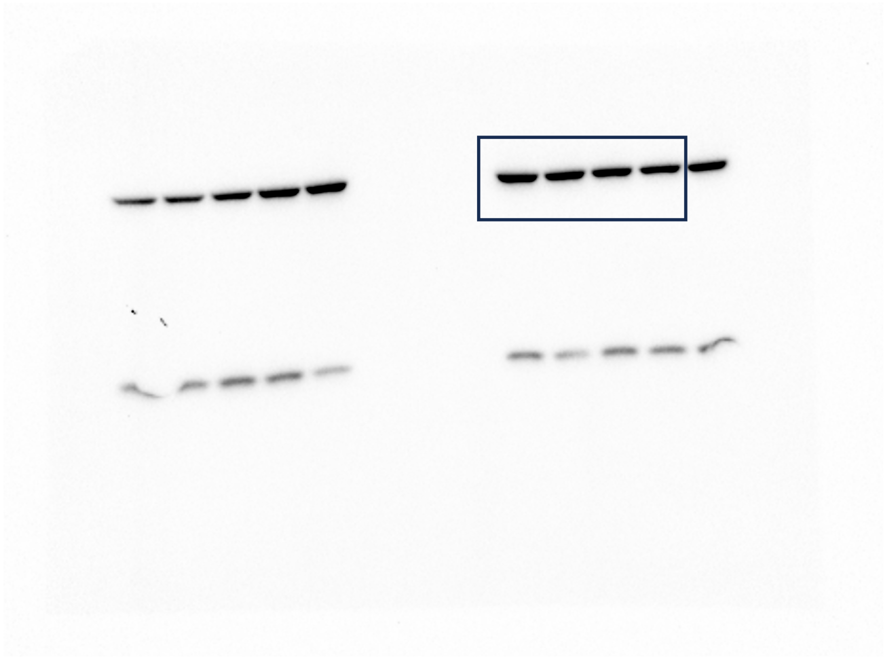

Hsp10

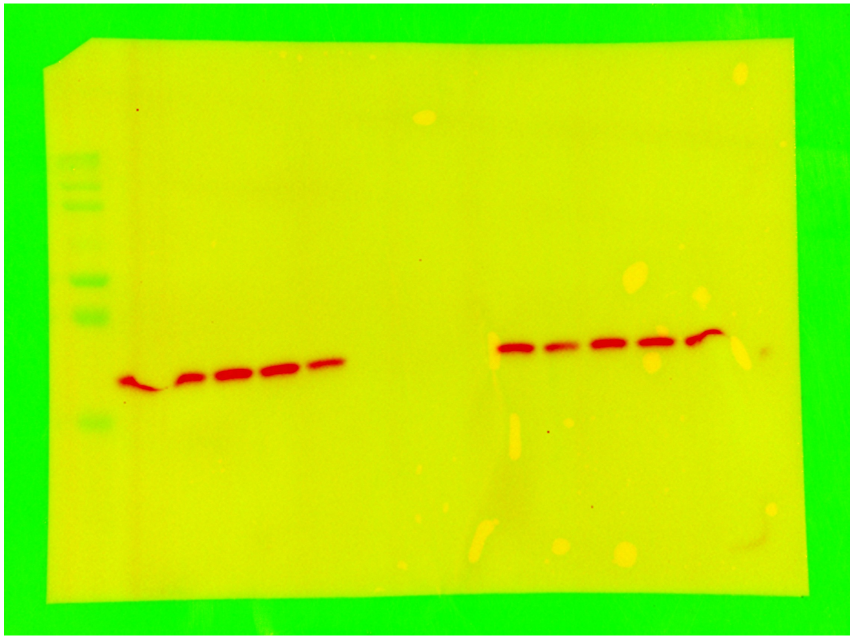

Actin

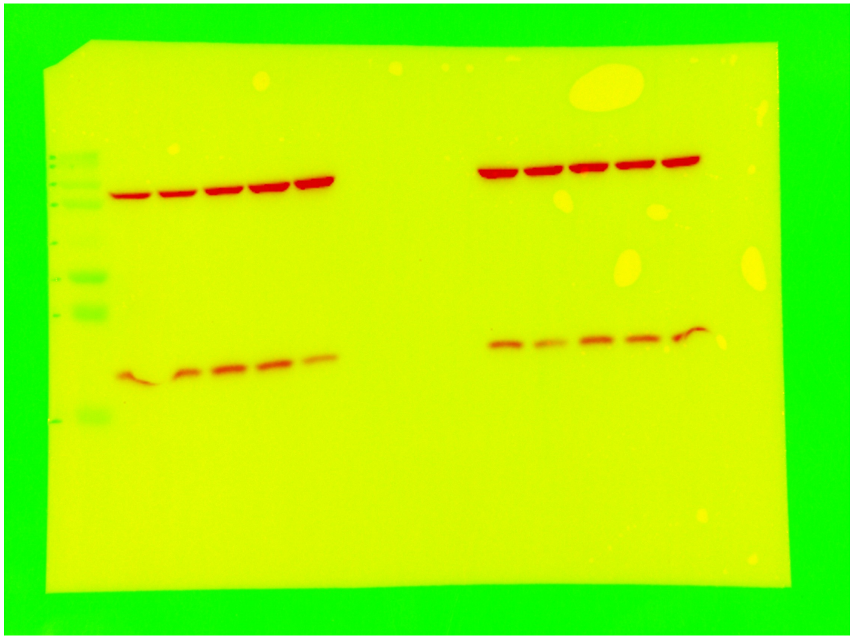

Hsp10

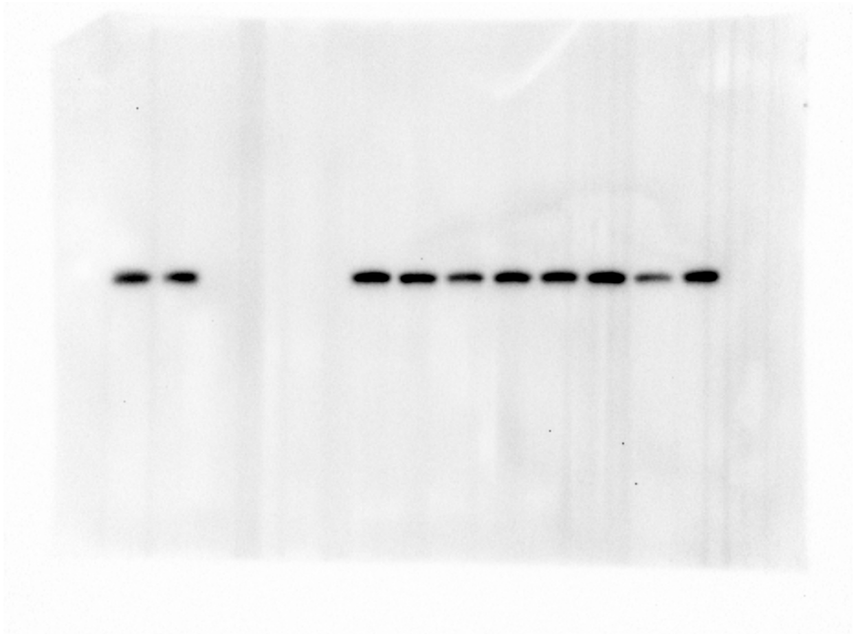

Actin

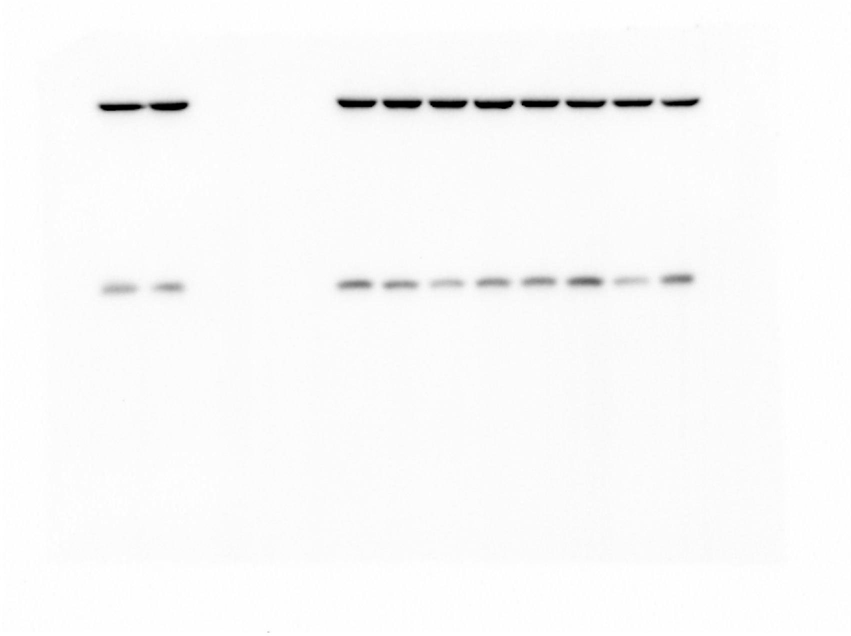

Hsp10

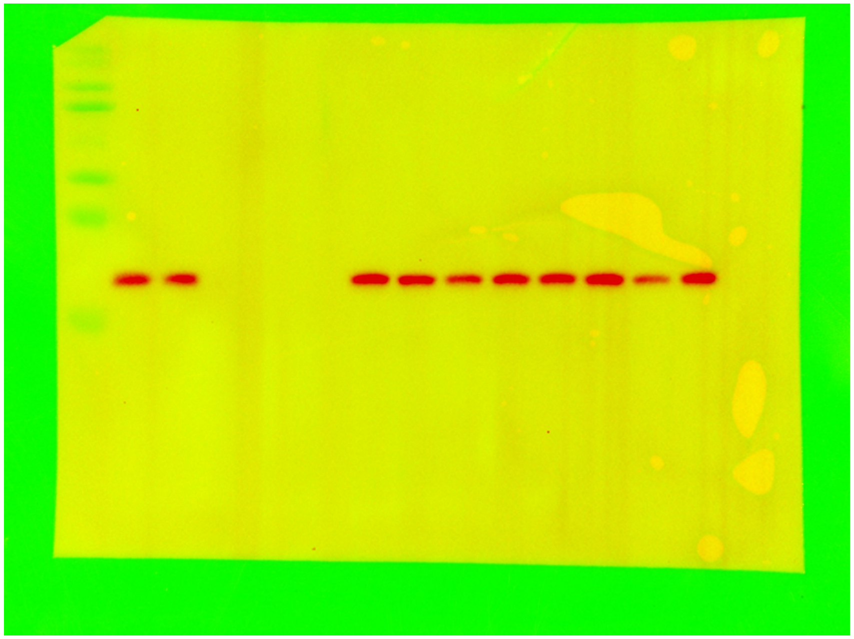

Actin

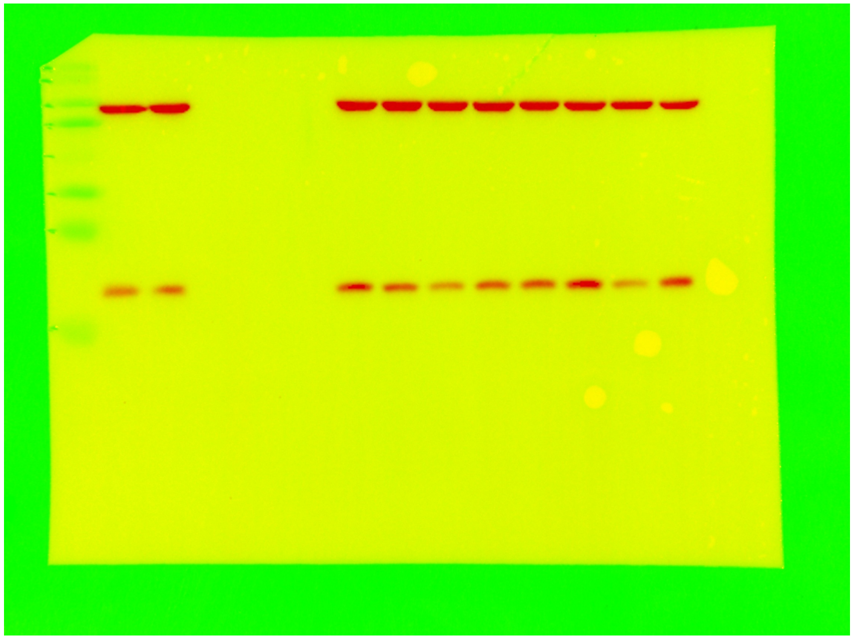

FoxO3a

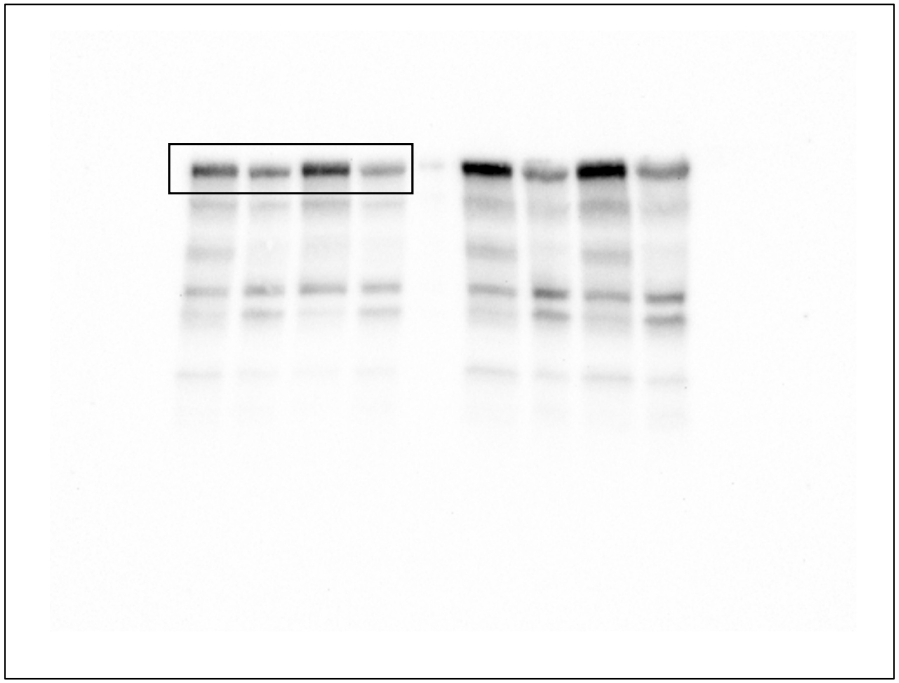

Actin

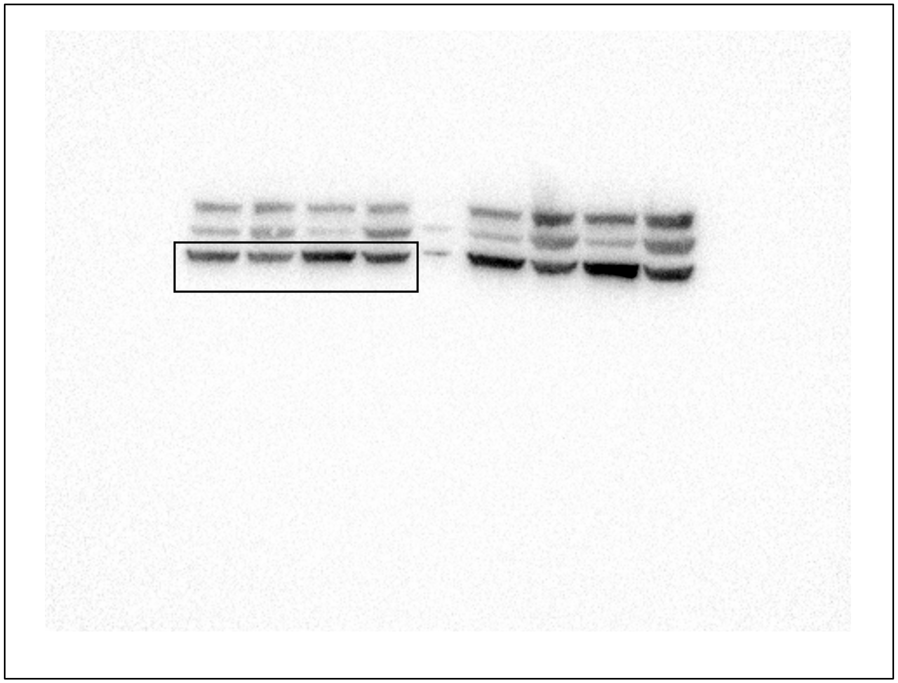

FoxO3a

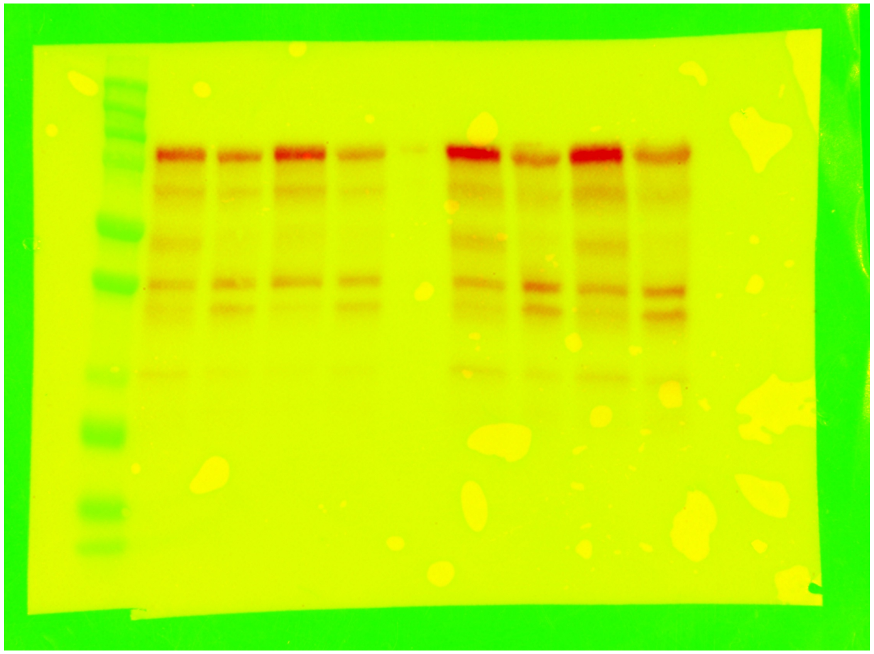

Actin

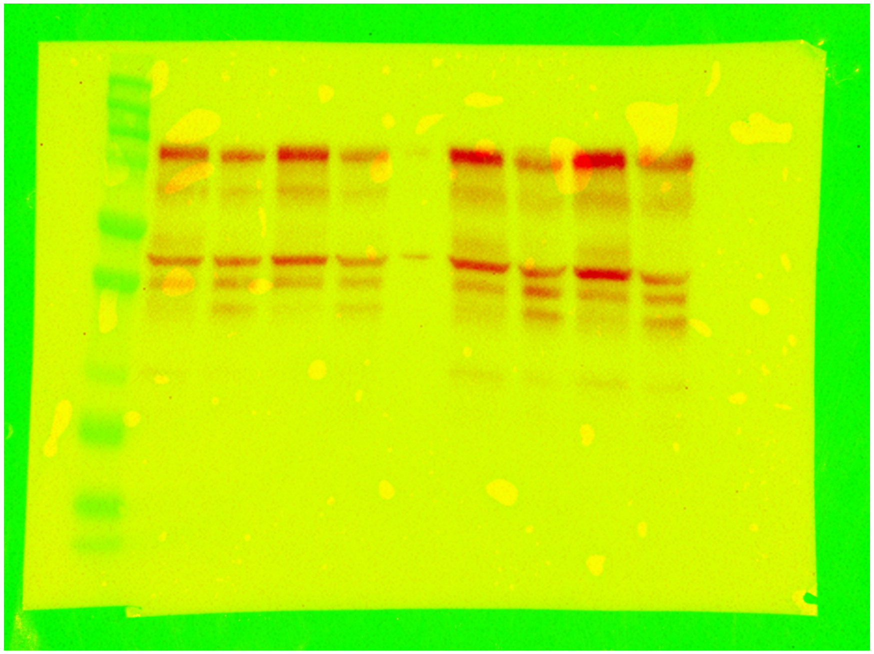

Acetyl FoxO3a

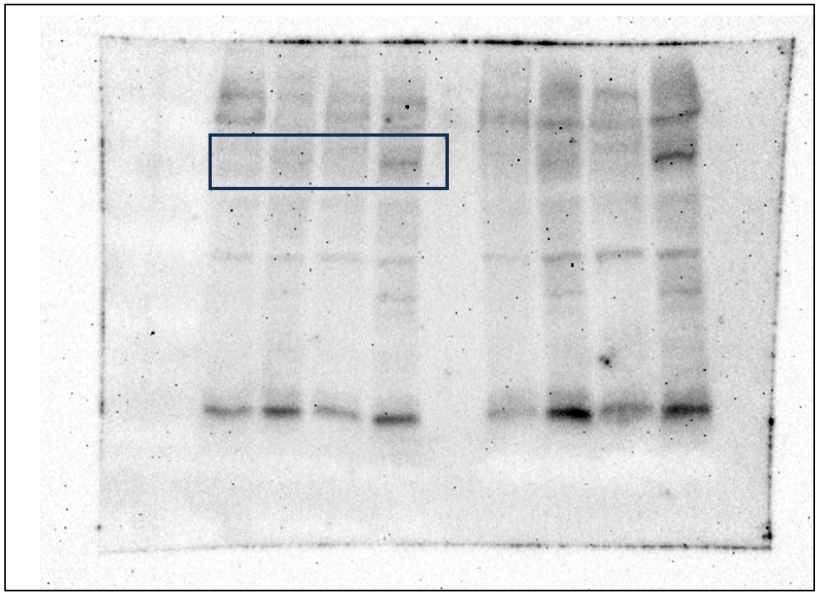

Actin

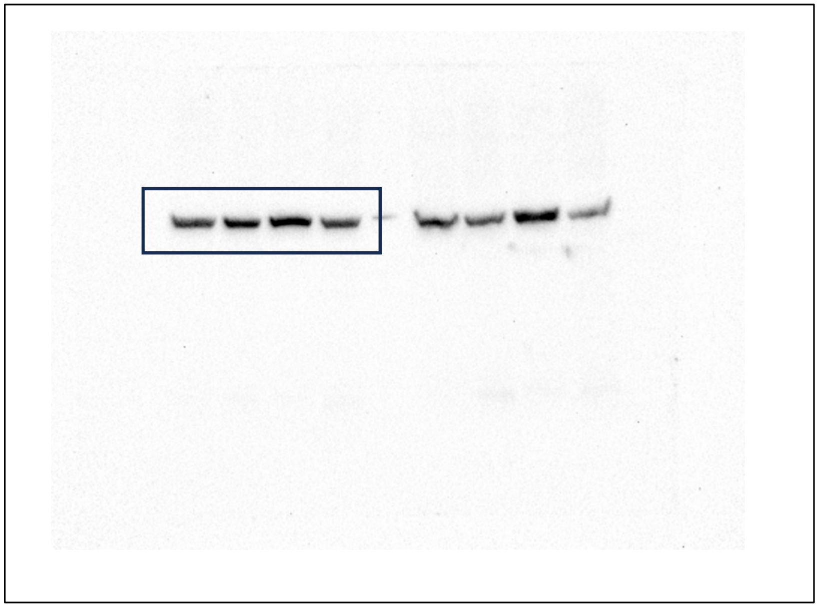

Acetyl FoxO3a

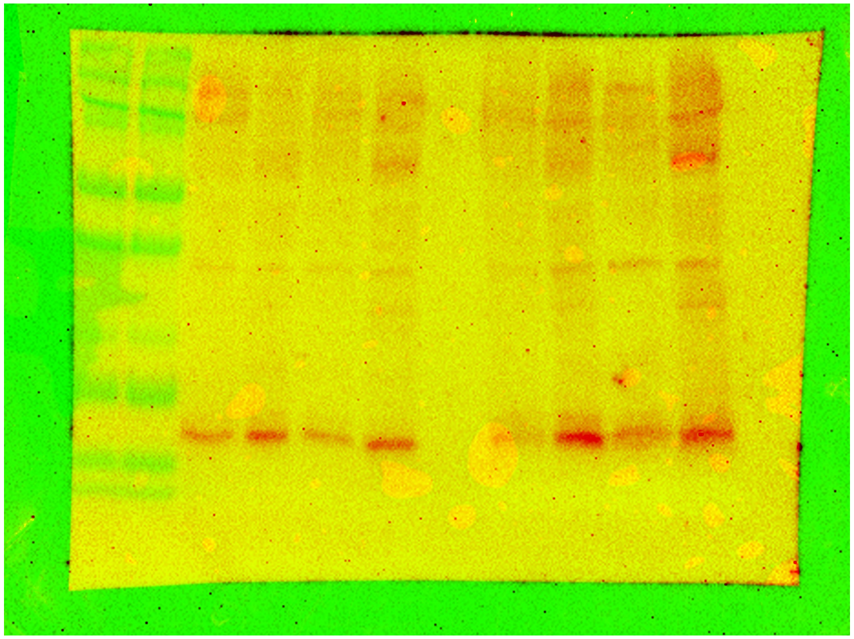

Actin

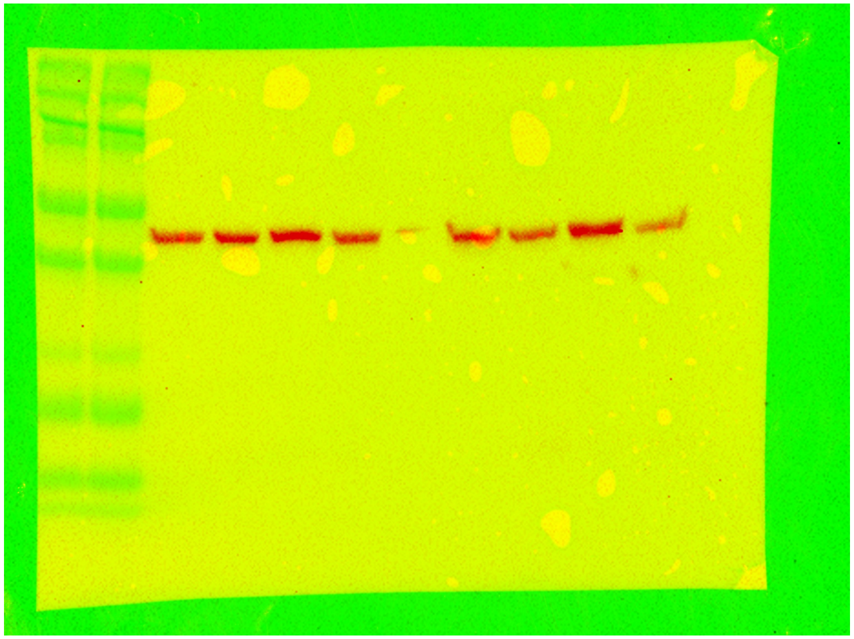

Uncropped Gels from Figure 5B

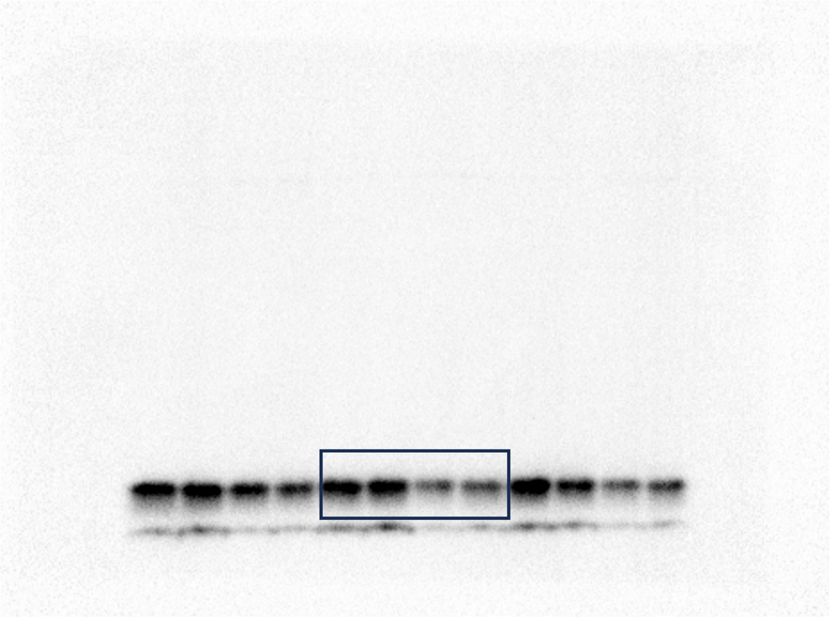

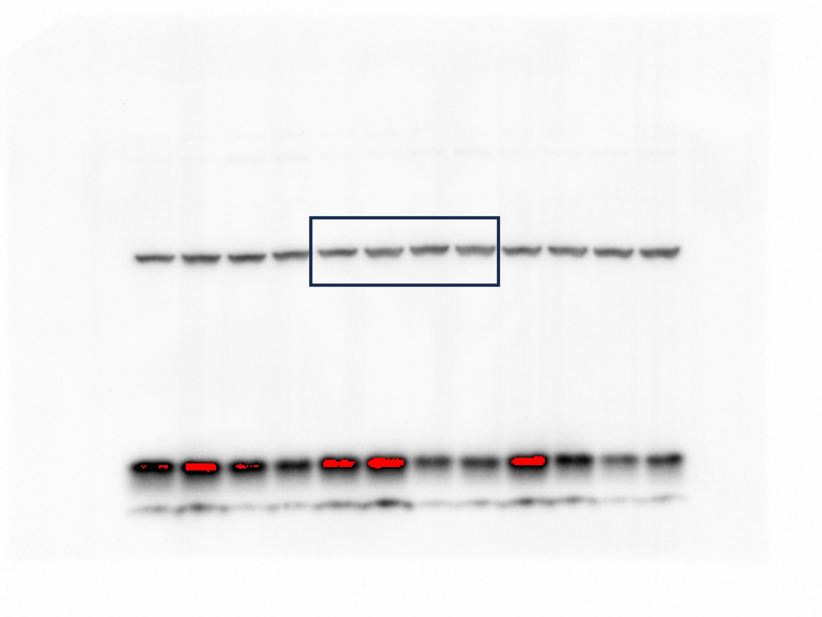

TOMM20

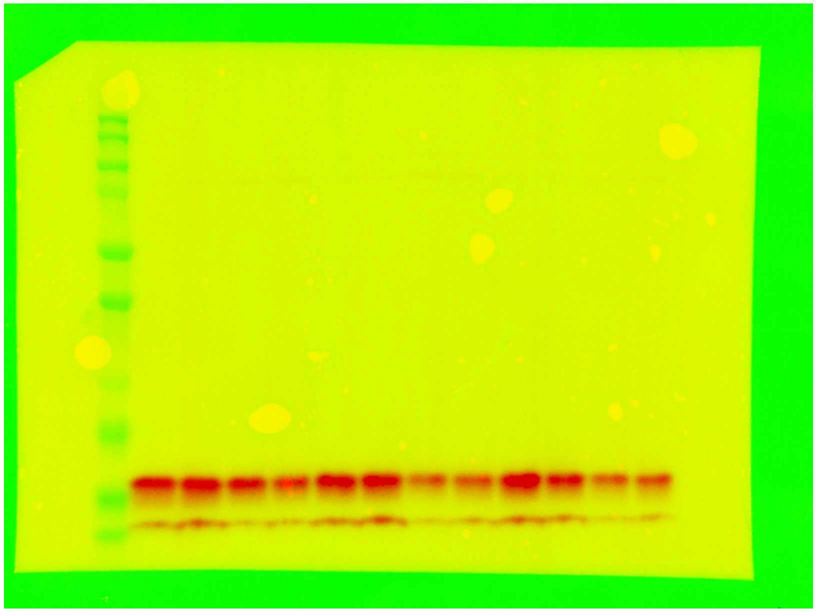

Actin

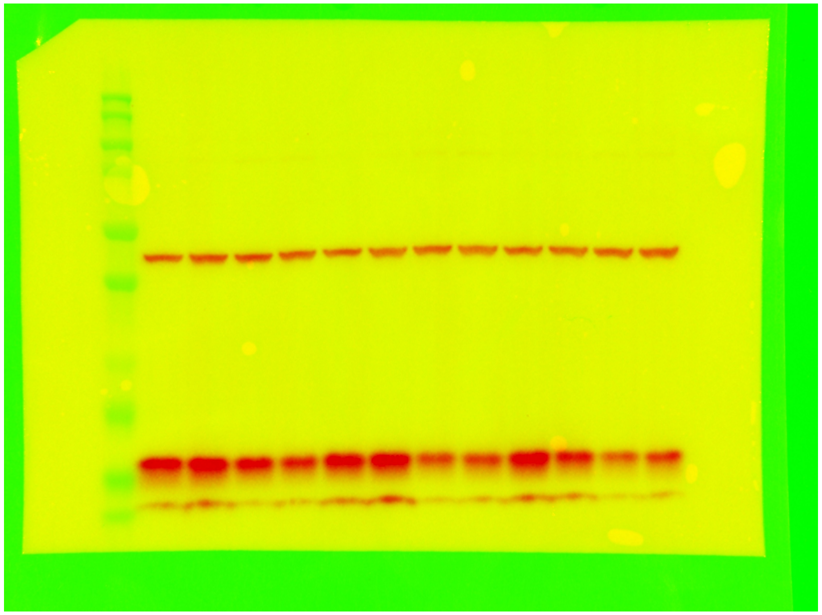

Parkin

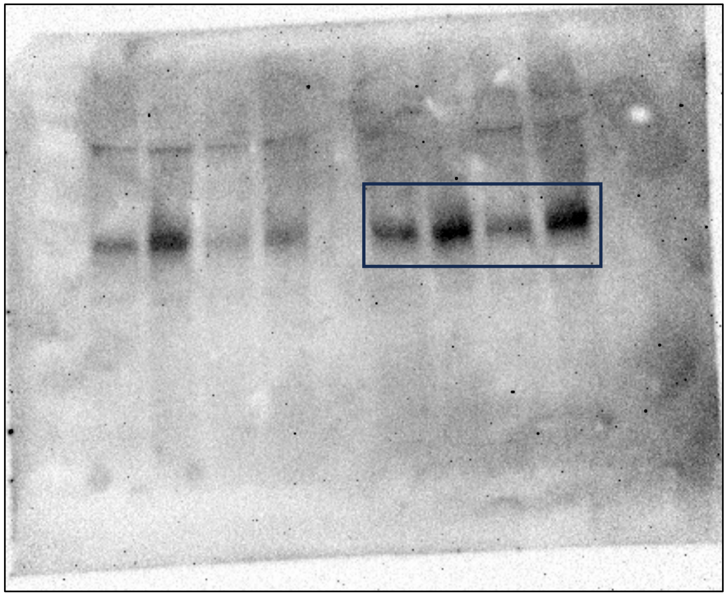

Actin

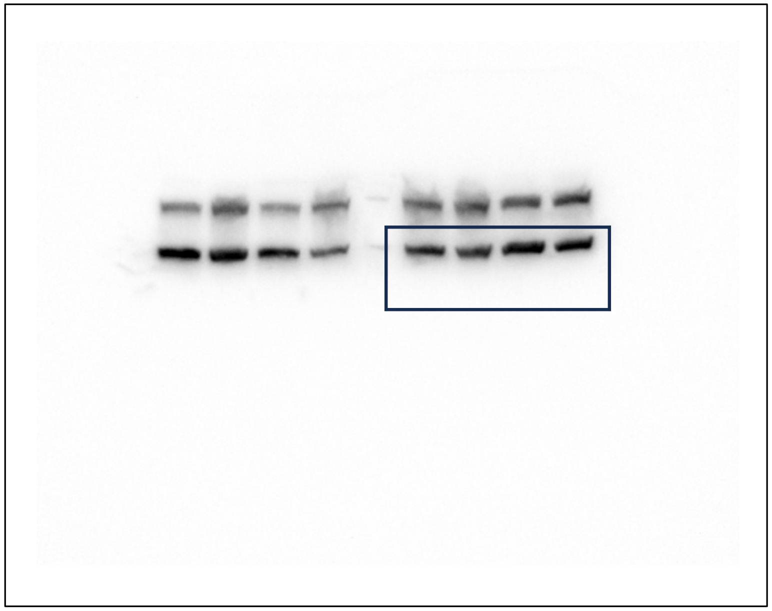

Parkin

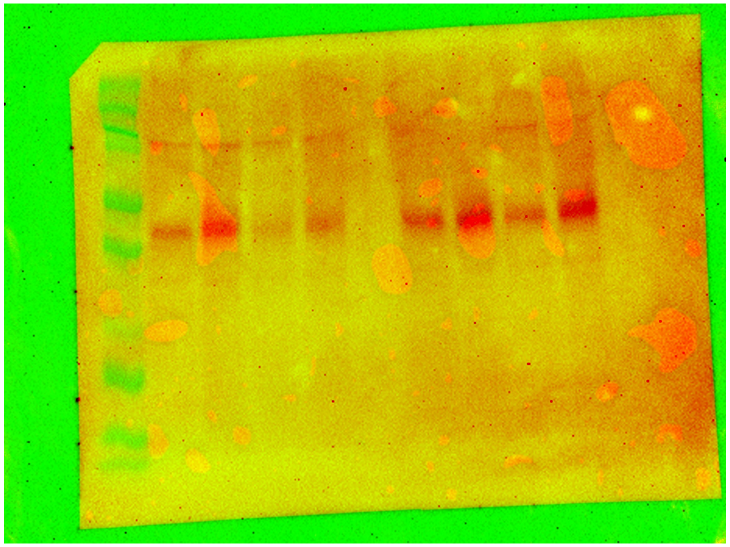

Actin

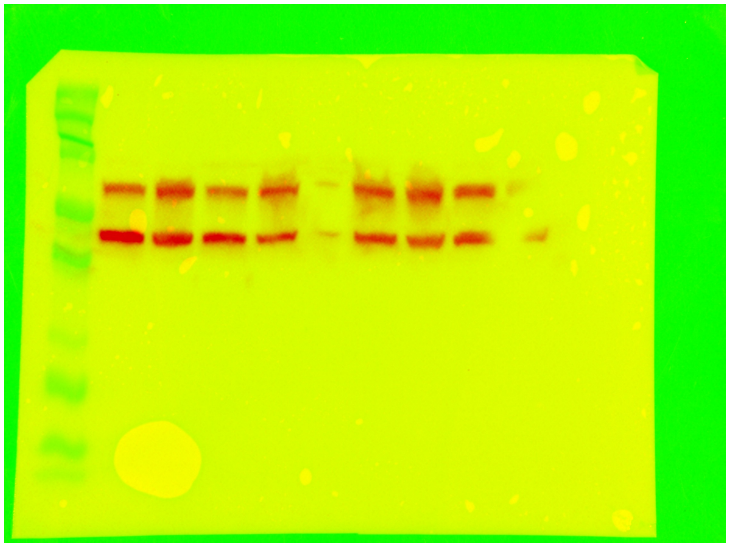

LC3B

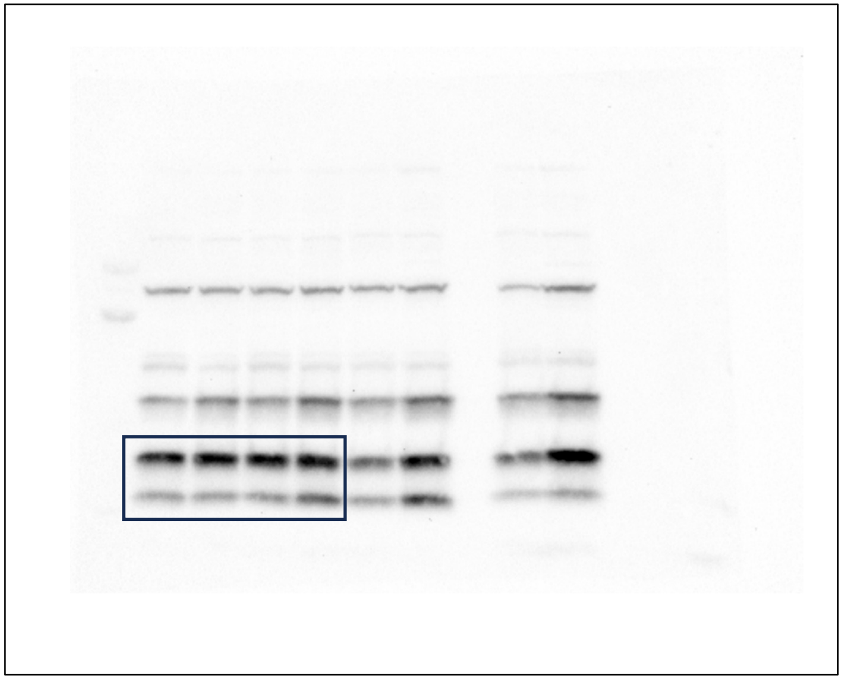

Actin

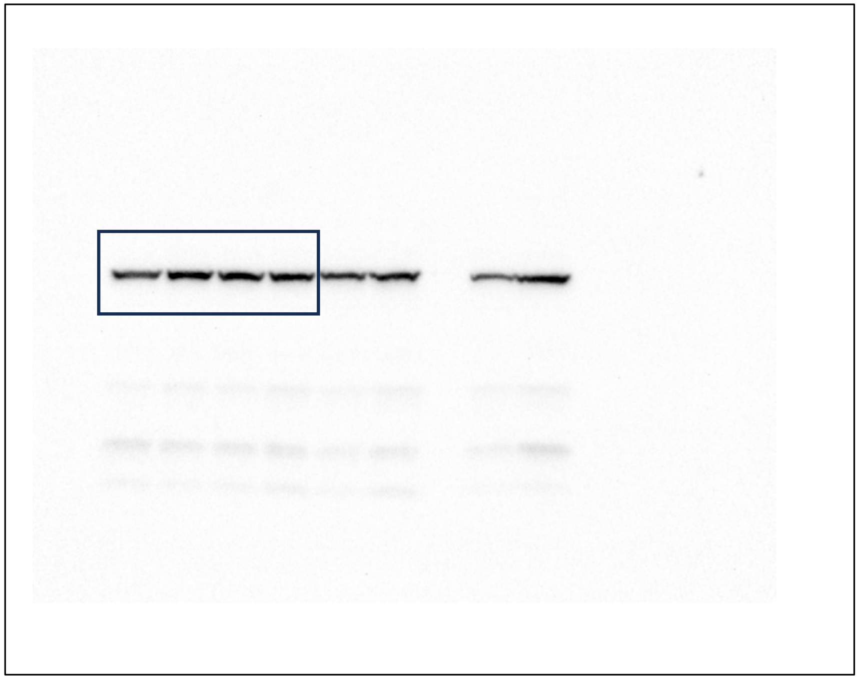

LC3B

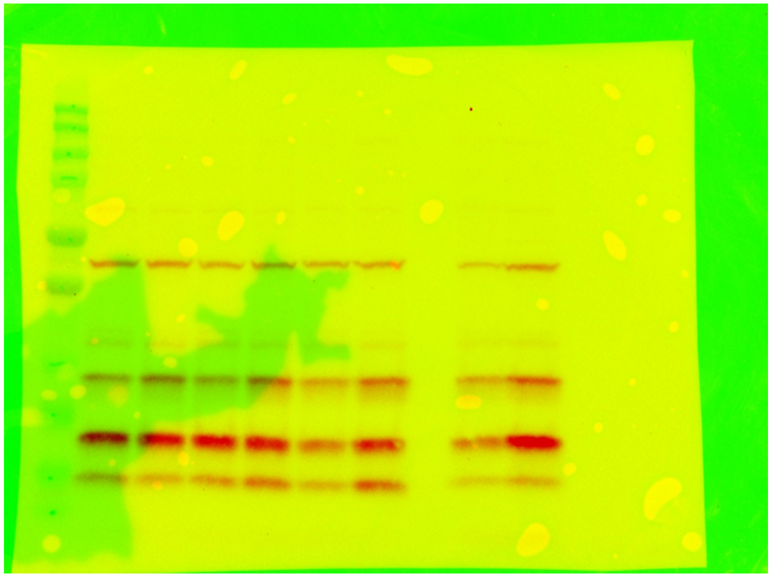

Actin

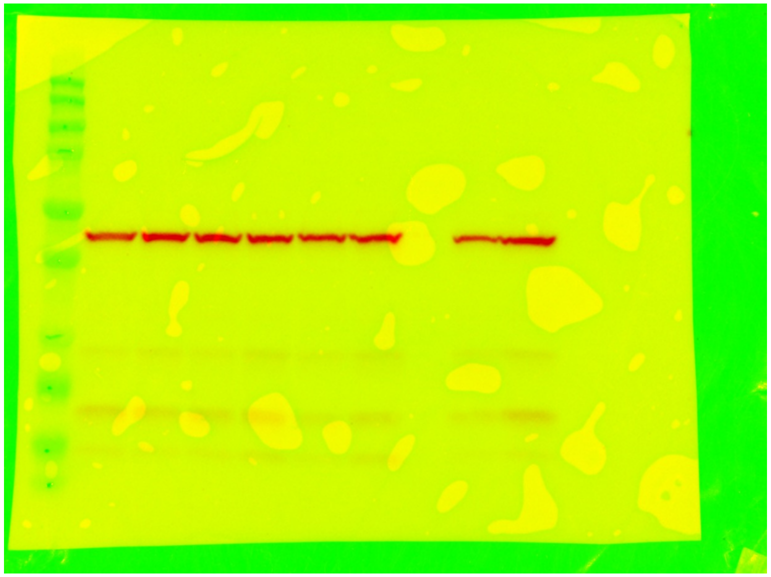

Supplement: Supplementary file 2 [file DataSheet1.pdf]
